# Immune Checkpoint Inhibitors and Survival Outcomes in Brain Metastasis: A Time Series-Based Meta-Analysis

**DOI:** 10.3389/fonc.2020.564382

**Published:** 2020-10-20

**Authors:** Xingjiang Hu, Hui Yu, Yunliang Zheng, Qiao Zhang, Meihua Lin, Jialei Wang, Yunqing Qiu

**Affiliations:** ^1^Research Center of Clinical Pharmacy, The First Affiliated Hospital, College of Medicine, Zhejiang University, Hangzhou, China; ^2^Zhejiang Provincial Key Laboratory for Drug Evaluation and Clinical Research, The First Affiliated Hospital, College of Medicine, Zhejiang University, Hangzhou, China; ^3^Department of Medical Oncology, Fudan University Shanghai Cancer Center, Shanghai, China; ^4^Department of Oncology, Shanghai Medical College, Fudan University, Shanghai, China

**Keywords:** immune checkpoint inhibitor, brain metastasis, survival, prognosis, meta-analysis

## Abstract

Immune checkpoint inhibitors (ICIs) have shown potential to improve the prognosis of patients with brain metastasis (BM) caused by advanced cancers. However, controversies still exist in regard to its survival benefits. In the present work, a time series-based meta-analysis based on the phase I/II/III trials and observational studies were performed to investigate the differences in mortality of ICI-treated BM patients. A number of public library databases, including MEDLINE, EMBASE, OVID, and COCHRANE, were systemically searched by March 2019. The quality of included studies was evaluated by the Newcastle-Ottawa Scale (NOS) scoring. Outcome measures here established were mortality and progression-free survival (PFS) at different follow-up endpoints. Survival rates and curve data were pooled for further analysis. To detect the data heterogeneity, subgroup analyses were conducted according to tumor and ICI types. Eighteen studies, 6 trials, and 12 controlled cohorts were assessed, involving a total of 1330 ICI-treated BM patients. The 6-month survival rate and PFS were 0.67 (95%CI: 0.59–0.74) and 0.36 (95%CI: 0.24–0.49), respectively. According to the tumor type (melanoma, NSCLC, and RCC), subgroup analyses indicated that melanoma presented the lowest survival rates among the three groups here selected. In regard to the type of ICIs, the anti-CTLA-4 combined with the anti-PD-1/PD-L1 showed the best survival outcome among these groups. The 12-month survival rate and PFS showed a consistent pattern of findings. In the long-term, the 24-month survival rate and PFS were 0.20 (95%CI: 0.12–0.31) and 0.18 (0.05–0.46) in BM patients. Hence, ICI therapy may be associated with an improved prognosis of BM patients. Nevertheless, current research presented a limited study design. Multicenter randomized trials may later assist in validating ICI-based therapies for a better outcome of BM patients.

## Introduction

Brain metastasis (BM) is a well-known devastating complication of advanced malignancies that frequently leads to substantial morbidity and mortality. BM occurs in 20–40% of adult patients affected by solid primary tumors outside the central nervous system (CNS), and its incidence has constantly increased (1, 2). This rise on BM incidence is possibly related to the increase on its detection by advanced imaging tools, such as magnetic resonance imaging (MRI), as well as more effective therapies capable of controlling extracranial systemic disease, thus causing later manifestations of the disease (3). Malignancies such as melanoma, lung, renal cell, and breast cancers are the most common types that are able to metastasize to the brain (4). BM patients may show the symptoms such as headache, altered mental state, focal weakness, sensory change, or seizures. Still, most BM patients have no symptoms and may be diagnosed secondarily to comprehensive staging that includes the CNS. Due to the aggressivity of cancer, BM patients have high mortality of 81–95% and usually die due to neurocognitive sequels (5, 6).

At present, although local treatment modalities including neurosurgical resection, stereotactic radiosurgery (SRS), stereotactic ablative radiotherapy (SABR), or whole brain radiotherapy (WBRT), have been the major methods of BM treatment, the optimal choice(s) of treatment are still controversial. Current recommendations have been mostly based on the disease prognosis, in addition to the number, size, and location of the brain lesions (1). Due to the poor tumor control during BM treatment, WBRT usually leads to radio-resistance. In addition, the toxicity of this therapeutic approach has been well-demonstrated by the modality of neurocognitive decline, mainly attested by memory loss and impaired executive function (7). On the contrary, SRS has overcome this limitation by considering higher doses of radiation, so a better control on tumor expansion has been reported, in the range of 73–90%. In addition, SRS has been often used as an adjuvant therapy during surgical resection. Thus, SRS can achieve the effective local control (LC) of established BM but its application is limited to the number of detected metastases (8). Surgery alone can improve the symptomatic burden of BM, however, LC failure rate can be as high as 59% during the follow-up examination for 2 years (9). For many patients, BM treatment consists of radiation and/or surgical resection. Systemic therapies are achieving more importance, since an increasing number of drugs have been approved for the therapy of advanced cancers by showing improved overall survival (OS) and progression-free survival (PFS). Nevertheless, the accessibility of these drugs into the brain are frequently obstructed by blood brain barrier (BBB), thus compromising their efficacy (10). Chemotherapy has presented limited curative effect in decreasing the tumor burden of CNS. Novel kinase inhibitors targeting oncogene-driven NSCLC [for instance, involving mutated epidermal growth factor receptor (*EGFR*) or gene rearrangement of anaplastic lymphoma kinase (*ALK*)], as well as melanoma (due to *BRAF* mutation) have presented therapeutic activities, but these are often brief and/or incomplete, so BM patients are jeopardized by a very poor prognosis (11).

Nowadays, immune checkpoint inhibitors (ICIs) have revolutionized the treatment of BM, covering different lines of treatment, with promising efficacy outcomes and tolerable safety performance. Major developments in the studies of immunotherapies have shown improved survival of patients with advanced cancers (12). These achievements have changed the standard of care for BMs as shown in the updated guidelines recently (13).

A number of immunotherapies have used ICIs to block the interaction of immune checkpoints and then enable an immune response against tumor cells. T-lymphocyte-associated protein 4 (CTLA-4) as well as programmed cell death protein 1 (PD-1) and its ligand (PD-L1) have been the most evaluated and approved checkpoint inhibitors (14). Many retrospective studies, usually originated from single institution, have explored the safety and efficacy between SRS and ICI in the treatment of BMs, with multiple prospective trials currently being planned or underway. While these trials are evolving, clinicians are required to make medical decisions based on limited information. Hence, here we attempted to pool published data focused on BM to evaluate the safety and efficacy of ICI based on different endpoint survival rates, PFS, and adverse events.

So far, a limited number of meta-analyses have directly evaluated the efficacy of ICIs. In our current evaluation, we have examined the putative predictive value of routinely collected data to further guide the selection of BM patients for ICI treatment as second and/or later lines of therapy. In this regard, a quantitative meta-analysis that combines information from similar endpoint results could serve as a rational approach to evaluate overall effects and to investigate sources of heterogeneity. Still, some reports have presented indirect survival data with Kaplan–Meier curves and, therefore, have not provided detailed information for each particular endpoint. After the development of GetData Graph Digiter 2.24 software (http://getdata-graph-digitizer.com/), the digitization and extraction of data have been possible (15). Hence, we have been able to extract data, at specific time points, and bring the observation periods accordingly with the GetData software.

Herein, we presently retrospect the contemporary peer-reviewed literature containing high-quality data related to the clinical management of multiple BM cases. Based on this data review, emerging recommendations for patient management are discussed meanwhile future areas of interest for clinical investigation are also proposed. Given the advances in systemic BM therapies, a critical evaluation of the outcome(s) for all BM patients is warranted.

## Methods

### Data Sources

To conduct the current meta-analysis, Preferred Reporting Items for Systematic Reviews and Meta-analysis (PRISMA) guidelines were followed (16). Database search using MEDLINE, EMBASE, OVID, COCHRANE library database, the American Society of Clinical Oncology (ASCO), and the European Society for Medical Oncology (EMSO) conference proceedings (through January 2013) was performed. The search strategy included the MeSH terms and text words: (“brain metastases” or “cerebral metastasis” or “intracranial metastases” or “central nervous system” or “CNS metastasis”) and (ipilimumab or nivolumab or pembrolizumab or atezolizumab or anti-PD-1 or anti-PD-L1 or anti-CTLA-4 or “immune checkpoint inhibitors”). References of the included studies as well as related reviews were checked manually. All additional studies of potential interest were retrieved for further analyses. If trials were serially published, only the most recent and complete clinical report was considered for evaluation. Studies in non-English language and/or involving animals or minor subjects were excluded. Reviews, abstracts, case reports, conference presentations, editorials, and expert opinions were also excluded to minimize potential publication bias and duplicated results. In addition, academic conference and clinical trial registration website were also researched due to some ongoing clinical research. Since the current data were obtained from previous published studies, no ethical approval and patient consent were required.

### Study Selection

Two co-authors (HXJ and YH) screened the title and abstracts of all retrieved citations. The full texts related to these citations were assessed according to the pre-established inclusion criteria (i.e., population, intervention, comparison, outcome, and study design [PICOS]). All relevant articles underwent evaluation for eligibility, by the same investigators, in an unbiased manner.

The selection criteria were defined according to the PICOS framework. Hence, this meta-analysis included the following: (i) population: participants with histologically confirmed BMs; (ii) intervention: patients submitted to ICI alone or after radiation treatment; (iii) comparison: ICI alone; (iv) outcome: survival data should be sufficient and also contain a critical endpoint (OS and PFS), with follow-up period ≥1 month; and (v) study design: cohort or phase I/II/III trials.

### Data Extraction

Survival outcome data were extracted from texts, tables, and figures. Data from analyzed articles were extracted independently by two co-authors (XH and QZ). Disagreements were resolved through discussion or consultation with a third author. The following characteristics were collected in each study: (i) first author, (ii) year of publication, (iii) country of origin, (iv) study design, (v) recruitment period, (vi) duration of follow-up, (vii) number of research centers, (viii) demographic and clinical information of the participants, and (ix) the incidence of adverse events. If articles showed survival data indirectly by using Kaplan–Merier curves, Getdata Graph Digitizer was applied to process and extract time-specific data. Trial name, tumor type, number of participants, ICI type, mOS (95%CI), mPFS (95%CI), clinical trial stage, and research period were collected from academic abstracts or prospective studies still under development.

### Data Analysis

Statistical analysis was performed with R 3.5.1. Stratification analyses were conducted for the following groups: (i) 6-, 12-, and 24-month OS and PFS; (ii) 6- and 12-month OS and PFS, grouped by the tumor location; and (iii) 6- and 12-month OS and PFS, grouped by the type of ICIs. Treatment-related toxicity was summarized and coded using the Common Terminology criteria for Adverse Events (CTCAE) Version 4.03. Heterogeneity across the studies was assessed by *Q*-test and *I*^2^ statistics. Heterogeneity was considered statistically significant when *P* < 0.05 or *I*^2^ > 50%. A random-effect model was used when evidence of significant heterogeneity was detected, otherwise a fixed-effect model was applied. Pooled OS and PFS were calculated using proper algorithms. A sensitivity analysis was also performed using a 1-study-removed analysis. Kaplan–Meier estimates of survival data were analyzed upon grouping according to the type of ICI. *P* < 0.05 was regarded as statistically significant. All *P*-values were two sided.

### Quality Assessment

The strength of evidence for each outcome was evaluated by two independent assessors (ML and YZ), using the Grading of Recommendations, Assessment, Development, and Evaluations (GRADE) approach. In this case, a table summarizing the findings was presented to properly identify and annotate the certainty of all pooled outcomes. Thereafter, each study was retrieved by the Newcastle-Ottawa Scale (NOS) to account for selected criteria, such as selection, comparability, and outcome, to evaluate the quality of the experimental design (17).

The outcome measure was defined as survival at different follow-up endpoints. Short-survival measurement was assessed from reported follow-up periods (6- and 12-month), while long-term survival measurement was assessed at 24-month follow-up. To detect the influence of ICI and tumor types, subgroup analyses were performed at 6- and 12-month period. Subgroup analyses were also conducted to explore possible sources of heterogeneity.

### Bias Assessment

Publication and small-study bias were assessed after the generation of a funnel plot and then retrieved for asymmetry Egger's linear regression and Begg's correlation tests to investigate suspect asymmetry for small-study bias. Sensitivity analysis by exclusion was performed for all outcomes to evaluate the risk of single-study bias.

## Results

### Literature Search

**Figure 1:**
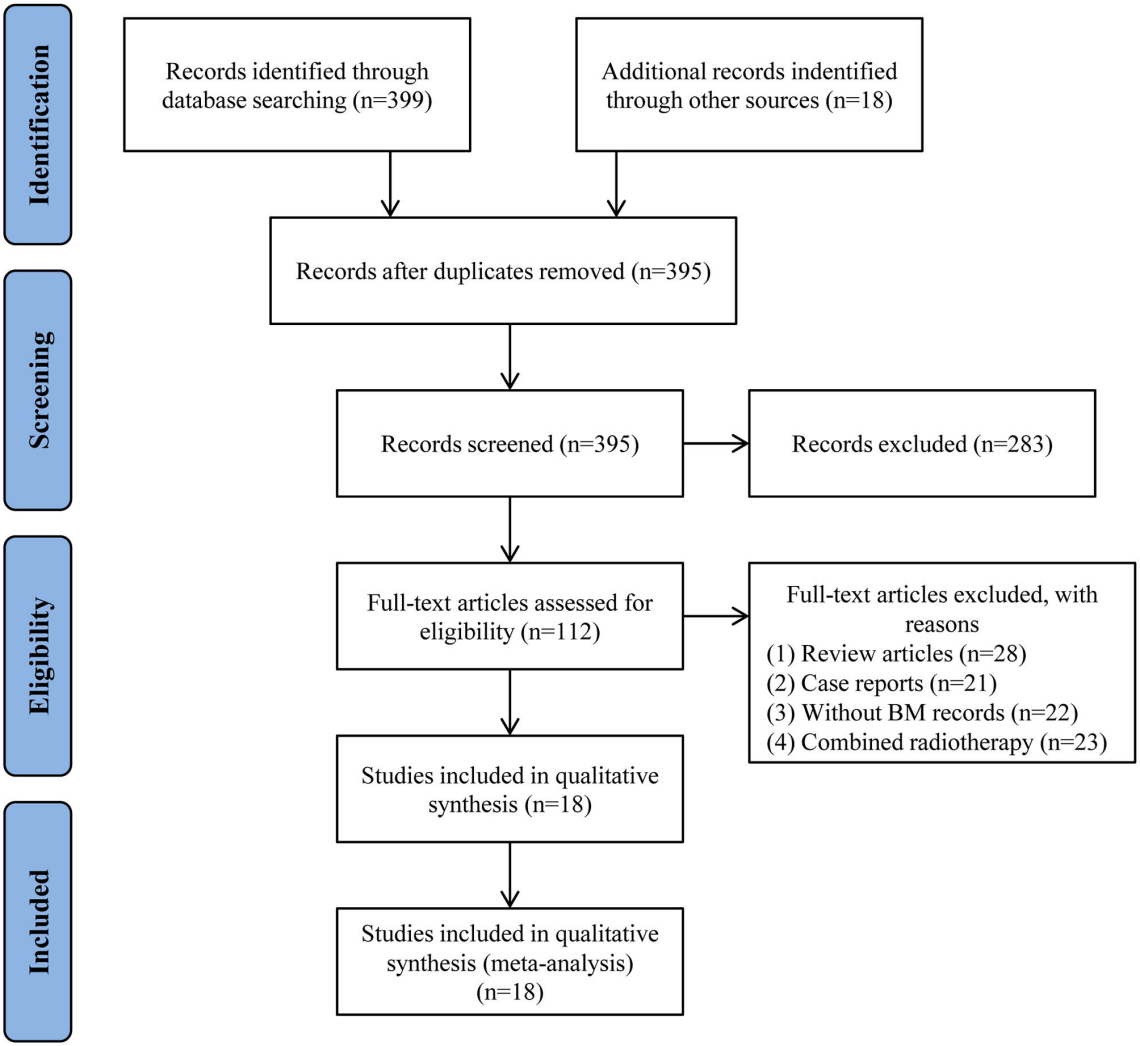
Flow chart of study selection process.

According to the pre-established screening procedures (Figure 1), 417 potentially relevant articles were identified in respective databases and other sources. A total of 22 duplicate articles were excluded. Then 283 articles were excluded by reviewing the titles and abstracts. After retrieval of full-text articles, 94 articles were further excluded due to (i) review articles, (ii) case reports, (iii) without BM records, or (iv) combined radiotherapy. Finally, 18 articles, published between the year of 2014 and 2019, were eligible for inclusion in this study (18–35).

### Study Characteristics

Table 1 lists the major characteristics of the studies presently selected. All studies enrolled patients within the past decade, while most of them were published in the past 3 years. Among the 18 included reports, 12 were related to retrospective studies (18, 20–23, 25–29, 32, 33), one was a phase I trial (24), four were phase II trials (19, 30, 31, 35) and another one was a phase III trial (34). Seven reports contained multicenter studies (21, 24, 29, 30, 32–34). All included studies were of high-quality with scores ranging from 7 to 10, according to the NOS criteria.

**Table 1:** Baseline characteristics of including studies for meta-analysis.

**Study**	**Type BMs included**	**Total No. of patients (*n*)**	**No. of patients treated with radiotherapy**	**Article style**	**No. of centers**	**Cancer location**	**Drug designation**	**Dosage**	**Study period**	Score*
Queirolo et al. (18)	Asymptomatic	146	6	Retrospective study	NR	Melanoma	Ipil	3 mg/kg every 3 weeks	NR	7
Qian et al. (20)	Treated with Gamma Knife SRS	22	22	Retrospective study	NR	Melanoma	Ipil/Pemb	Ipil: either 3 or 10 mg/kg (*n* = 19); Pemb: either 2 or 10mg/kg every 2 or 3 weeks (*n* = 3)	2007–2015	9
Goldberg et al. (19)	Untreated	36	22	Phase II trial	1	NSCLC + Melanoma	Pemb	10 mg/kg every 2 weeks	2014.03–2015.05	8
Theurich et al. (21)	NR	41	40	Retrospective study	4	Melanoma	Ipil	3 mg/kg every 3 weeks	2011.03–2014.11	7
Chen et al. (26)	NR	30	30	Retrospective study	1	NSCLC + RCC + Melanoma	Ipil/Pemb/Nivo	NR	2010–2016	7
Yusuf et al. (25)	NR	6	6	Retrospective study	1	Melanoma	Ipil	3 mg/kg	2008–2015	8
Williams et al. (24)	NR	11	11	Phase I trial	2	Melanoma	Ipil	3 mg/kg (*n* = 3); 10 mg/kg (*n* = 8)	2012.10–2014.08	9
Cowey et al. (27)	NR	41	NR	Retrospective study	1	Melanoma	Pemb	1.9 ± 0.2 mg/kg	2014.09–2016.09	8
Patel et al. (23)	NR	20	20	Retrospective study	1	Melanoma	Ipil	3mg/kg	2009–2013	7
Parakh et al. (22)	All	66	21	Retrospective study	1	Melanoma	Nivo/Pemb	Nivo: 3 mg/kg every 2 weeks (*n* = 6); Pemb: 2 mg/kg every 3 weeks (*n* = 60)	2012.10–2016.03	9
Long et al. (30)	Cohort A: Asymptomatic, untreated	35	0	Phase II trial *subgroup*	4	Melanoma	Nivo plus Ipil	Nivo 1 mg/kg combined with Ipil 3 mg/kg every 3 weeks for 4 doses, followed by Nivo 3 mg/kg every 2 weeks	2014.11–2017.04	10
	Cohort B: Asymptomatic, untreated	25	0				Nivo	3 mg/kg every 2 weeks		
	Cohort C: Symptomatic	16	9				Nivo	3 mg/kg every 2 weeks		
Spigel et al. (31)	Asymptomatic, treated	13	NR	Phase II trial *subgroup*	1	NSCLC	Atez	1,200 mg every 3 weeks	2013.05–2017.03	8
Kluger et al. (35)	Asymptomatic, untreated	23	17	Phase II trial *subgroup*	1	Melanoma	Pemb	10 mg/kg every 2 weeks	2014.03–2015.08	9
Tawbi et al. (32)	Asymptomatic	94	8	Retrospective study	28	Melanoma	Nivo plus Ipil	Nivo 1 mg/kg combined with Ipil 3 mg/kg every 3 weeks, followed by Nivo 3 mg/kg every 2 weeks	2015.02–2017.06	10
Gauvain et al. (29)	NR	43	NR	Retrospective study	2	NSCLC	Nivo	3 mg/kg every 2 weeks	2015.05–2016.08	8
Gabani et al. (28)	NR	192	192	Retrospective study	NR	Melanoma	Ipil	NR	2011–2013	7
Gadgeel et al. (34)	Asymptomatic and treated	61	55	Phase III trial *subgroup*	31	NSCLC	Atez	1,200 mg every 3 weeks	2014.11–2018.12	7
Crino et al. (33)	Asymptomatic	409	NR	Retrospective study	153	NSCLC	Nivo	3 mg/kg every 2 weeks	NR	9

*BMs, brain metastases; NSCLC, Non-small-cell lung cancer; NR, Not report; RCC, Renal Cell Carcinoma; Ipil, Ipilimumab; Nivo, Nivolumab; Pemb, Pembrolizumab; Atez, Atezolizumab*.

* *The quality of citation was assessed according to NOS (retrospective study and phase I, II trial) and Jadad score (phase III trial)*.

Moreover, most of the aforementioned studies consisted of patients with melanoma metastases (18–28, 30, 32, 35), while six reports were related to NSCLC metastases (19, 26, 29, 31, 33, 34), and one to renal cell carcinoma metastases (26). These studies involved a total of 1,330 BM patients, of which 459 have received radiotherapy. The most commonly used ICIs were anti-PD-1/PD-L1, which were applied in nine of the studies (19, 22, 27, 29–31, 33–35). Anti-CTLA-4 were administered in six of the studies (18, 21, 23–25, 28), and combined immunotherapies focusing on anti-CTLA-4 and anti-PD-1/PD-L1 were utilized in other four studies (30, 32).

### Short-term PFS in BMs

**Figure 2:**
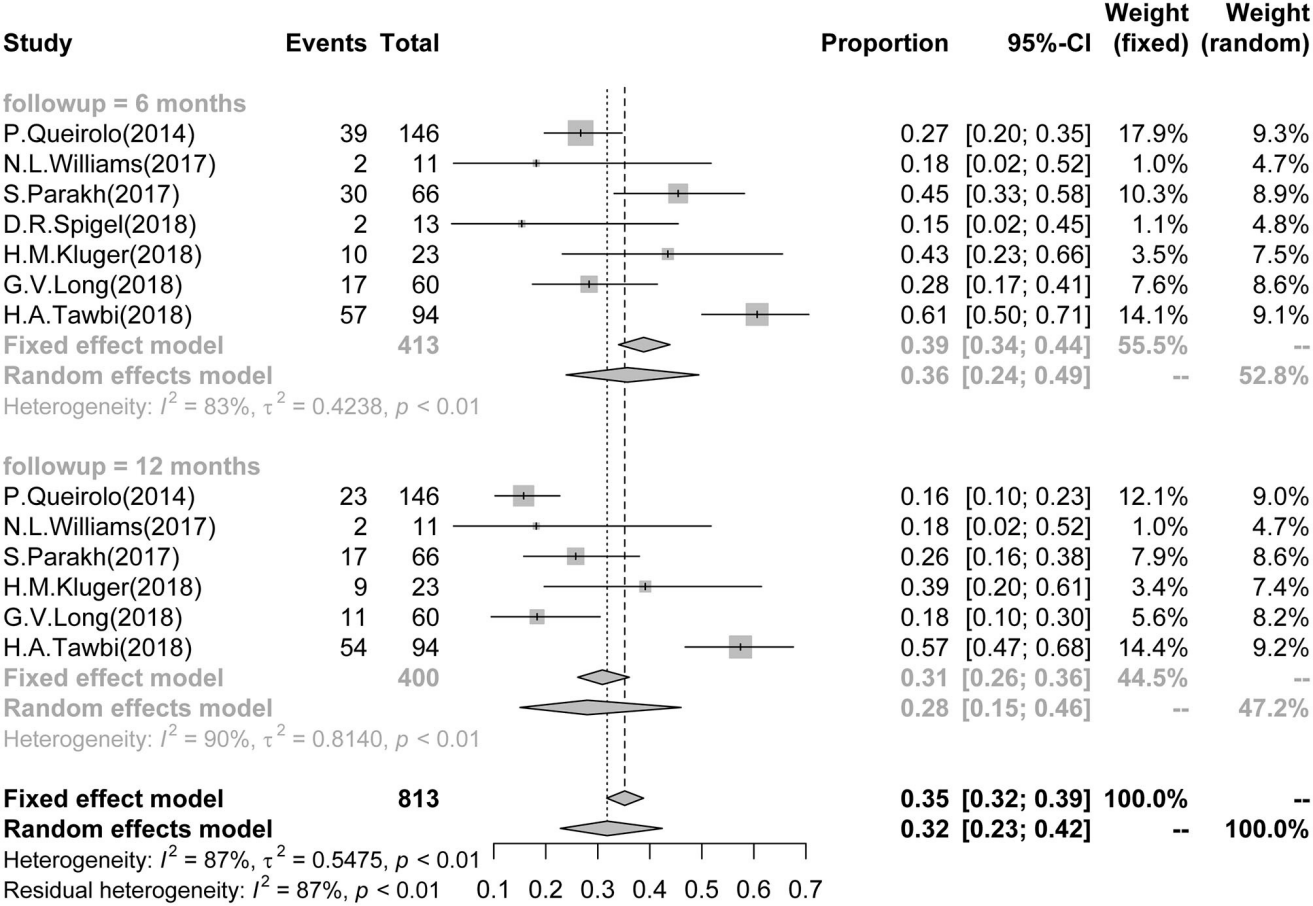
Association between ICIs and Short-term PFS in BM patients. Forest plots showing 6- and 12-month PFS in BM patients treated with ICIs. ICI, immune checkpoint inhibitor; BM, brain metastasis; PFS, progression-free survival.

Seven studies reported 6-month PFS data of BM patients, which involved a total of 413 patients. The pooled and calculated 6-month PFS ratio was 0.36 (95%CI: 0.24–0.49; *I*^2^ = 83%, *P* < 0.01; Figure 2). According to the tumor type, patients with melanoma had a higher PFS in subgroup analysis (Supplementary Figure 1). According to the ICI type, subgroup analysis showed that patients receiving treatment with combined anti-CTLA-4 and anti-PD-1/PD-L1 presented the highest PFS in all three groups [CTLA-4: 0.25 (0.18–0.33), *I*^2^ = 0%; PD-1/PD-L1: 0.33 (0.18–0.48), *I*^2^ = 63%; combined CTLA-4 and PD-1/PD-L1: 0.48 (0.24–0.73), *I*^2^ = 86%] (Supplementary Figure 2).

Six studies reported 12-month PFS of BM patients, involving a total of 400 patients. The 12-month PFS for these patients was 0.28 (95%CI: 0.15–0.46; *I*^2^ = 90%, *P* < 0.01; Figure 2). Only melanoma patients reported 12-month PFS, and no subgroup analysis was arranged by tumor type (Supplementary Figure 3). According to the ICI type, subgroup analysis showed that patients receiving treatment with combined anti-CTLA-4 and anti-PD-1/PD-L1 had the best survival outcome in all three groups [CTLA-4: 0.15 (0.09–0.21), *I*^2^ = 0%; PD-1/PD-L1: 0.25 (0.13–0.39), *I*^2^ = 56%; combined CTLA-4 and PD-1/PD-L1: 0.40 (0.11–0.74), *I*^2^ = 92%] (Supplementary Figure 4).

### Short-Term Survival in BMs

**Figure 3:**
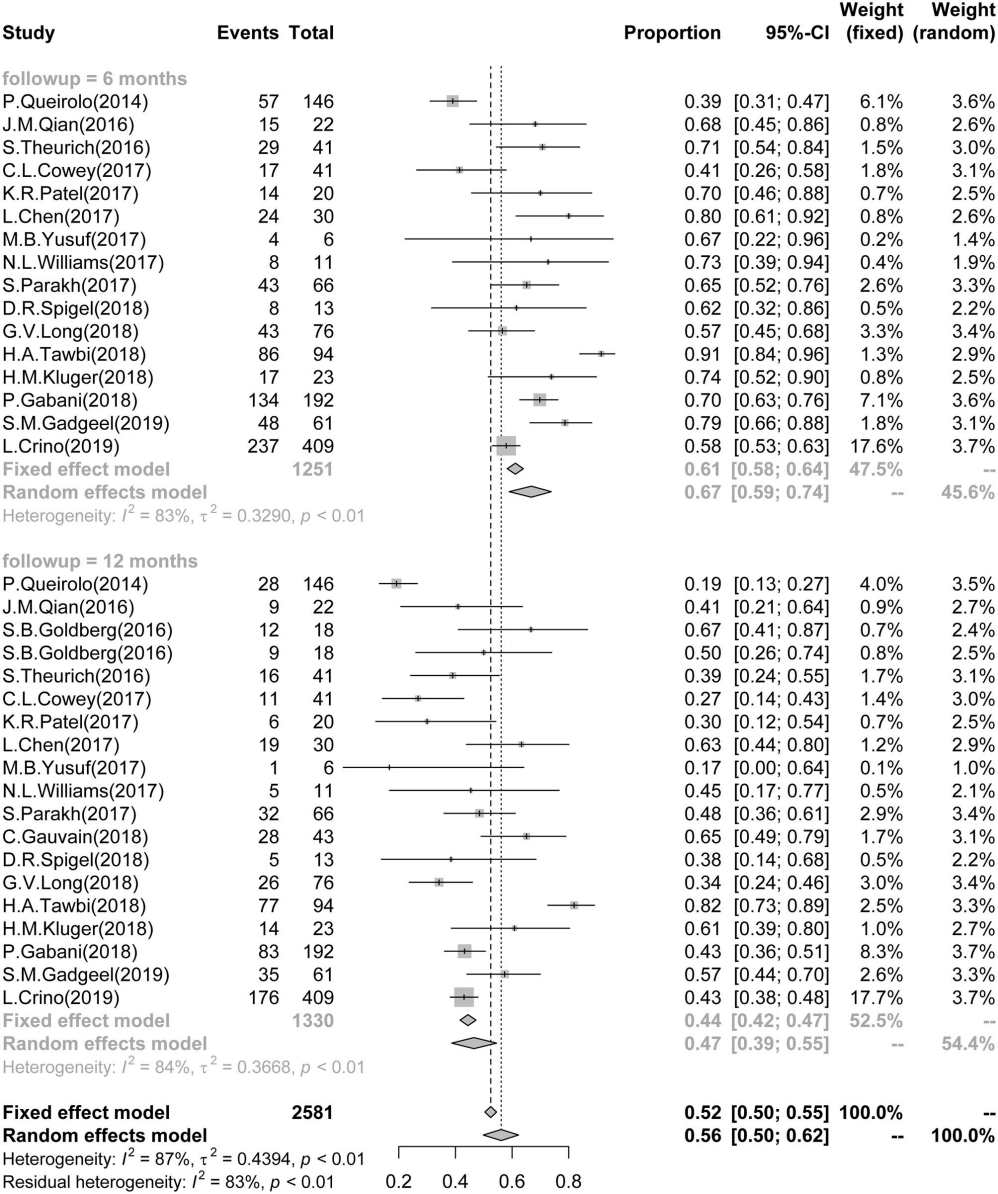
Association between ICIs and Short-term Survival in BM patients. Forest plots showing 6- and 12-month survival rate in BM patients, treated with ICIs. ICI, immune checkpoint inhibitor; BM, brain metastasis.

A total of 16 studies reported 6-month survival data and involved 1,251 patients. The pooled and calculated 6-month survival ratio was 0.67 (95%CI: 0.59–0.74; *I*^2^ = 83%, *P* < 0.01) (Figure 3). According to the tumor type, subgroup analysis showed that the patients with melanoma had the poorest survival in all three groups [melanoma: 0.66 (0.55–0.75), *I*^2^ = 85%; NSCLC: 0.67 (0.49, 0.80), *I*^2^ = 78%; NSCLC + RCC + melanoma: 0.80 (0.62–0.91)] (Supplementary Figure 5). According to the ICI type, subgroup analysis exhibited that patient treatment based on anti-CTLA-4 and anti-PD-1/PD-L1 provided the best survival outcome in all three groups [CTLA-4: 0.64 (0.47–0.79), *I*^2^ = 87%; PD-1/PD-L1: 0.62 (0.53–0.71), *I*^2^ = 68%; combined CTLA-4 and PD-1/PD-L1: 0.77 (0.38–1.00), *I*^2^ = 94%] (Supplementary Figure 6).

Eighteen studies reported 12-month survival data and involved 1,330 patients. The pooled and calculated 12-month survival ratio was 0.47 (95%CI: 0.39–0.55; *I*^2^ = 84%, *P* < 0.01) (Figure 3). Similarly to the 6-month survival data, subgroup analysis regarding the tumor type showed that the melanoma patients had the poorest survival in all three groups [melanoma: 0.43 (0.32–0.55), *I*^2^ = 87%; NSCLC: 0.51 (0.41, 0.62), *I*^2^ = 63%; NSCLC + RCC + melanoma: 0.63 (0.45–0.78)] (Supplementary Figure 7). According to the ICI type, subgroup analysis showed that the treatment with combined anti-CTLA-4 and anti-PD-1/PD-L1 provided the best survival outcome in all three groups [CTLA-4: 0.32 (0.20–0.46), *I*^2^ = 80%; PD-1/PD-L1: 0.49 (0.41–0.56), *I*^2^ = 64%; combined CTLA-4 and PD-1/PD-L1: 0.55 (0.06–0.98), *I*^2^ = 97%] (Supplementary Figure 8).

**Figure 4:**
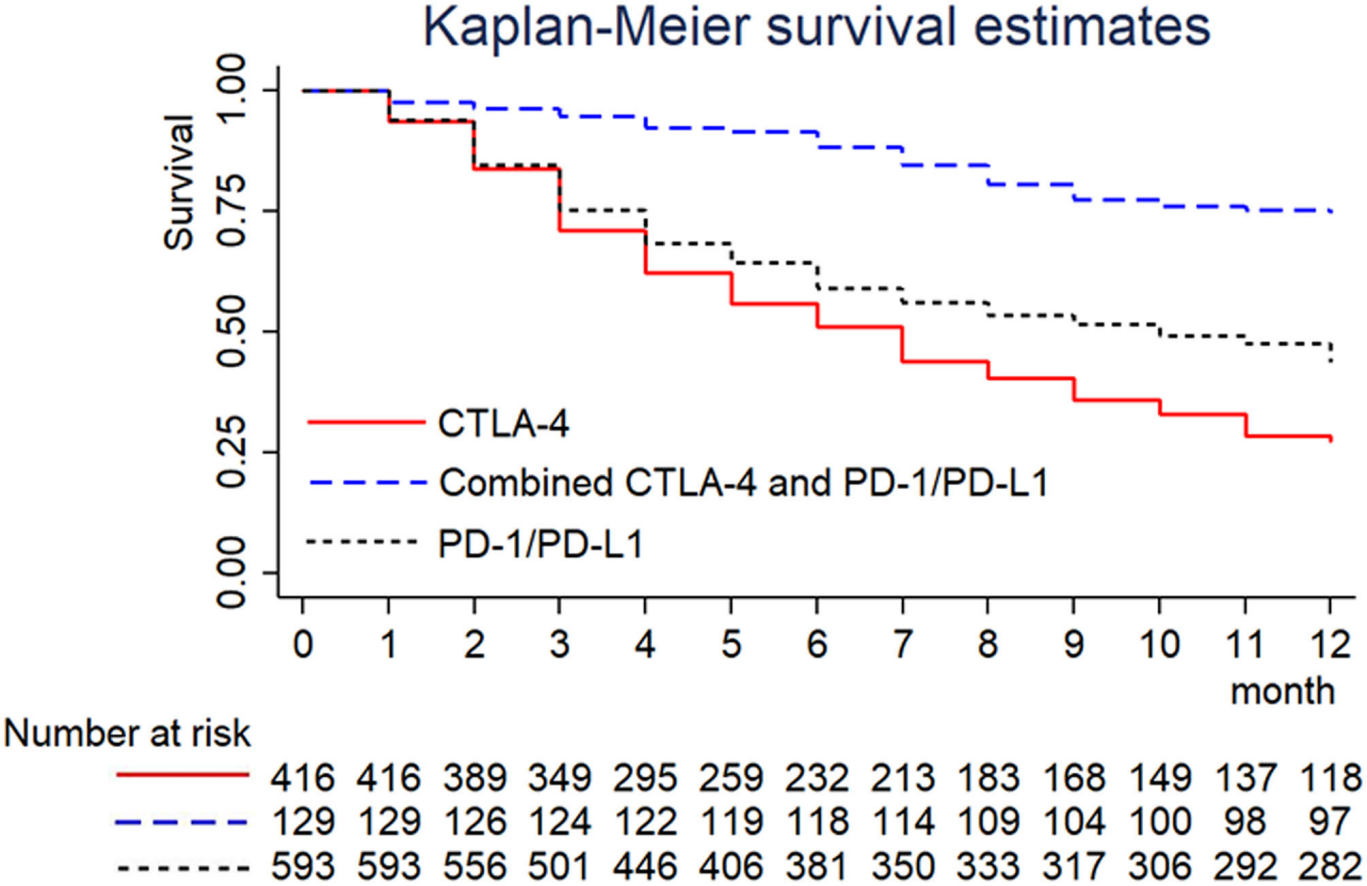
Kaplan–Meier curve of 12-month survival in BM patients treated with ICIs. Kaplan–Meier curve showing pooled 12-month survival in BM patients treated with ICIs.

Furthermore, 18 studies provided 12-month Kaplan–Meier curves of BM patients. Raw data were obtained from texts or extracted by digitizing graphs using the GetData software. The pooled Kaplan–Meier curves exhibited that the overall survival rate of patients treated with combined anti-CTLA-4 and anti-PD-1/PD-L1 was the highest in all three groups (Figure 4).

### Long-Term PFS and Survival in BMs

Five studies reported 24-month PFS data of BM patients and involved 340 patients. The pooled and calculated 24-month PFS ratio was 0.18 (95%CI: 0.05–0.46; *I*^2^ = 93%, *P* < 0.01; Supplementary Figure 9). The 24-month survival ratio was 0.20 (95%CI: 0.12–0.31; *I*^2^ = 91%, *P* < 0.01; Supplementary Figure 10). This last data set was retrieved from 14 studies and 1,215 patients.

### Meta-Regression and Sensitivity Analysis

The possible sources of heterogeneity among the included studies were investigated by meta-regression analysis. The screened sources were (i) year of publication, (ii) number of centers, (iii) number of participants, (iv) ICI types, (v) tumor types, and (vi) NOS/Jadad scoring. The results indicated that none of them (except year of publication for 6-month survival) explained the heterogeneity (*P* > 0.05). These results are shown in Supplementary Table 1.

All sensitivity analysis associated with the meta-analysis performed in this study suggested stable results (Supplementary Figure 11).

### Publication Bias

Funnel plots were generated for each particular outcome, to asymmetrically assess any publication bias. Hence, we evaluated the putative bias of the pooled rates and CIs by Begg's and Egger's tests. The publication bias was *P* > 0.05 in most of the clinical outcomes, according to both analytical tests (Supplementary Tables 2, 3 and Supplementary Figure 12).

### Academic Abstracts and Ongoing Prospective Studies

Based on the promising results, numerous ongoing clinical trials for ICIs have been pursued. In this context, six academic abstracts and five prospective studies are listed (Supplementary Tables 4, 5). All of the abstracts included NSCLC patients, while most of prospective studies were related to melanoma patients.

### Adverse Events

In the included studies, adverse events were reported inconsistently. Most studies reported the detailed names and grades of adverse events, four studies reported the total number of adverse events, one study did not report. For the reported adverse events, most were grades 1–2 and were well-tolerated and controlled, including fatigue, diarrhea, decreased appetite, and colitis. The detailed adverse events were summarized in Supplementary Table 6.

## Discussion

Historically, patients with melanoma-related BM have a poor prognosis (including fatal complications), with a median overall survival of 7–9 months (36). Clinical treatment of BM, originated from advanced cancers, is complex, and yet controversial. To date, topical treatments such as surgical resection, SRS and WBRT have become the mainstream. Nevertheless, these standard procedures can lead to serious complications and morbidity, due to stroke, radiation necrosis or cognitive dysfunction, with only a modest benefit in OS (37). The role of chemotherapy has been restricted since its penetration into the brain is widely limited by the BBB. In fact, even new-generation drugs with demonstrated efficiency in BM patients, such as osimertinib and lorlatinib, have still shown limited effects due to its specificity against particular mutations. In general, SRS or surgical resection have been recommended for patients with a single BM. For patients with 2 or 3 metastases, SRS is recommended for those who have good Karnofsky performance scores (KPSs). The role of sequential WBRT following SRS or surgical resection is still controversial. On this topic, a systematic review of treatment strategies for NSCLC patients with brain metastases has also been reported in detail (38).

Recently, advances on the field of immunotherapy have opened up a new therapeutic approach for BM patients (10). Unlike chemotherapy or targeted therapies, immunotherapeutic agents do not necessarily need to penetrate to the BBB to be clinically effective (39). ICIs are capable of enhancing anti-tumor immune responses against T cell regulatory pathways and have significant clinical efficacy against cancer (40). According to our knowledge, current results represent the most comprehensive meta-analysis evaluating the influence of ICIs in BM patients. This meta-analysis involved 18 studies that assessed the efficacy and safety of ICIs in two major solid tumors (i.e., melanoma and non-small cell lung cancer). The pooled results for the efficacy of ICIs in the treatment of distinct tumor types revealed that ICIs exhibit good disease progression [6-month survival: 0.67 (0.59–0.74); 12-month survival: 0.47 (0.39–0.55); 24-month survival: 0.20 (0.12–0.31); 6-month PFS: 0.36 (0.24–0.49); 12-month PFS: 0.28 (0.15–0.46); 24-month PFS: 0.18 (0.05–0.46)]. Subgroup analysis showed that patients with melanoma presented the poorest survival rate at 6- and 12-month follow-up. Goldberg et al. (19) have revealed that melanoma patients with BM had higher survival rate than NSCLC patients (67 vs. 50% survival rate for 12-month follow-up). These conflicting findings were possibly related to factors such as (i) uncontrolled design, (ii) small sample size of previous studies, and (iii) greater heterogeneity in the included studies. In fact, Queirolo et al. (18) have reported that the survival rates at 6- and 12-month were 39 and 19%, respectively (confirmed by sensitivity analyses). Co-treatment with anti-CTLA-4 and anti-PD-1/PD-L1 has presented the best outcome among three types of immunotherapy. The CheckMate 067 trial (12) of nivolumab, with or without ipilimumab (vs. ipilimumab monotherapy), using patients with only active extracranial melanoma have demonstrated that the combinatory therapy was associated with a higher proportion of clinical response, landmark PFS, and OS than nivolumab alone, although the study was not powered to establish statistical significance. Long et al. (30) have suggested that the presence of active BM might also be an additional baseline factor to take in consideration in the combinatory approach vs. monotherapy using nivolumab. Data from studies that may directly compare different ICIs in BM patients are still warranted.

Systemic immunotherapy has shown some preliminary but encouraging results for the treatment of BMs, changing the traditional paradigm of CNS immune privilege. The immune system recognizes tumor cell antigens by antigen presenting cells (APCs) and plays an important role in clearing oncogenic clonal cells. APCs activate T cells and subsequent T-cell mediated toxicity. In contrast, tumor cells can evade immune-promoting damage by expressing various immune checkpoint factors that promote self-tolerance and may inhibit effector T-cell function and proliferation.

In fact, although the CNS is no longer considered as an immune privileged site, it remains at least an immunodeficient environment. While tumor-infiltrating lymphocytes (TILs) have been clearly identified in BMs, those are in lower number when compared to systemic tumors and, moreover, the ratio of effector to regulatory T cells remains unknown. Besides, the traffic of T cells and APCs into and out of the CNS is more strictly regulated than in other tissues. The degree of blood tumor and blood brain barrier disruption is quite variable, depending on the disease, patient and even individual lesions in the same patient.

The utility of systemic therapies for the management of BMs has long been limited due to the consideration of the brain being “immunologically privileged.” However, some recent studies have demonstrated the presence of cytokine-responsive microglia and immune cells within the brain. In parallel to these discoveries, the anti-CTLA-4 agent ipilimumab was approved for the treatment of metastatic melanoma in 2011, followed by two PD-1 inhibitors pembrolizumab and nivolumab in 2014 (41).

The limited survival of BM patients have challenged the role of ICIs in the clinic since these patients have often been excluded from pivotal trials (42). The backdrop for this exclusion relies on the increased size of ICIs, which limits their ability to cross the BBB as well as the use of steroids to relieve symptomatic edema derived from BM, thus regulating the activity of the immune system and altering the risk of pseudo- and/or hyper-progression. Moreover, BM patients frequently need radiotherapy for local control, but safety data regarding the combination of cranial radiation plus ICIs remains sparse. This combinatory therapy seems to provide an opportunity to alleviate the stoppage on the immune system and then boost the abscopal response rates. Since limited studies have assessed the role of ICIs in BM patients, the generalizability of promising ICI data is still challenging.

Some previous studies also assessed the efficacy of specific ICIs (41, 43, 44), while our study differs from previous ones in several aspects. Firstly, some trials originally present in other meta-analyses were here excluded due to the stringent selection criteria. Secondly, several latest trial reports were updated and two interim reports from previous reviews were replaced by the final results of these trials. Specifically, these reports mostly evaluated therapeutic efficacy by comparing ICIs plus SRS vs. SRS alone. None of these studies reported the benefit of ICIs directly. Thirdly, the subgroup analysis of different ICI types was performed to explore the benefits of distinct treatment. Lastly, unlike other analytical studies, the OS and PFS data were compared point-to-point with the support of Getdata software.

ICIs have revolutionized the clinical landscape toward treatment of advanced cancers, but this approach has commonly excluded the BM patients from their pivotal trials. Normally, the daily clinical practice always imposes the use of ICIs in advanced cancers with BM, considering the promising survival time and duration of response. Surprisingly, a limited number of prospective trials have included BM patients but have barely reported the efficacy and/or safety of ICIs. The available data has been restricted to small retrospective or prospective series that show comparable efficacy to those of pivotal trials. Since most BM patients have received radiotherapy at some points of treatment, it is expected that the understanding of the interplay between radiotherapy and immunotherapy will be particularly interesting.

Of note, some disadvantages of this current meta-analysis should be noted. Firstly, the sample sizes of three individual trials were relatively small and may have generated false-negative or false-positive results due to random error. Secondly, most of included work were related to retrospective studies, potentially leading to some bias and weaker evidence than RCTs. Lastly, potential language bias should be considered since the studies presently assessed were those published in English language only. As systemic treatments improve and the lifespan of patients with metastatic diseases prolong, the number of BM patients may continuously increase, bringing greater unmet needs to patients with advanced cancers. Available evidence suggests that systemic immunotherapy has a promising efficacy toward untreated BM, thus supporting the integration of BM patients in immunotherapy-based clinical trials.

Admittedly, our meta-analysis has some limitations. Nevertheless, given that ICIs are novel medical procedures for the medium and advanced cancer patients, some clinical trials have been undergoing across the globe, such as “NCT02460068 (NIBIT-M2),” “NCT02374242 (ABC),” and others. Thus, more data related to the efficacy of ICIs in different populations will be acquired in the near future.

## Conclusion

In summary, ICIs may be associated with an improved prognosis for BM patients. However, current literature was limited by study design. The results of this systematic review confirm the need for additional clinical trials aiming the use of ICIs in BM patients.

## Data Availability Statement

All datasets presented in this study are included in the article/Supplementary Materials.

## Author Contributions

YQ and JW: study design and paper revision. XH and HY: data collection and statistical analysis. XH and QZ: paper writing. ML and YZ: data quality control. All authors: approved the submitted version of the manuscript.

## Conflict of Interest

The authors declare that the research was conducted in the absence of any commercial or financial relationships that could be construed as a potential conflict of interest.

## Abbreviations

ALK: anaplastic lymphoma kinase
ASCO: American Society of Clinical Oncology
BBB: blood brain barrier
BM: brain metastasis
BRAF: B-rapidly accelerated fibrosarcoma
CNS: central nervous system
CTCAE: Common Terminology criteria for Adverse Events
CTLA-4: T-lymphocyte-associated protein 4
EGFR: epidermal growth factor receptor
EMSO: European Society for Medical Oncology
GRADE: Grading of Recommendations, Assessment, Development and Evaluations
ICI: immune checkpoint inhibitors
LC: local control
MRI: magnetic resonance imaging
NOS: Newcastle-Ottawa Scale
NSCLC: non-small cell lung cancer
OS: overall survival
PD-1: programmed cell death protein 1
PD-L1: programmed cell death protein ligand
PICOS: population, intervention, comparison, outcome, and study design
PFS: progression-free survival
PRISMA: Preferred Reporting Items for Systematic Reviews and Meta-analysis
SABR: stereotactic ablative radiotherapy
SRS: stereotactic radiosurgery
WBRT: whole brain radiotherapy.
