# Supplementary material for: Immune Checkpoint Inhibitors and Survival Outcomes in Brain Metastasis: A Time Series-Based Meta-Analysis
Source: Front Oncol. 2020 Oct 20;10:564382. doi: 10.3389/fonc.2020.564382 (PMC7606910; doi:10.3389/fonc.2020.564382)
Supplement: Supplementary file 1 [file Data_Sheet_1.zip › Supplementary materials/Supplementary Table 1 meta regression.docx]

**Supplementary table 1: Meta regression for each outcome of included studies**

| **Outcome** | **factors** |  | **Coef.** | **Std. Err.** | ***t*** | ***P*** |
| --- | --- | --- | --- | --- | --- | --- |
| OS for 6-month | year of publication | year | 0.07 | 0.03 | 2.46 | 0.029 |
|  |  | _cons | -136.78 | 55.93 | 2.45 | 0.029 |
|  | total sample | sample | 0.00 | 0.00 | 0.55 | 0.592 |
|  |  | _cons | 0.70 | 0.07 | 10.47 | <0.001 |
|  | number of center | center | 0.00 | 0.00 | 1.14 | 0.284 |
|  |  | _cons | 0.64 | 0.04 | 14.85 | <0.001 |
|  | ICI type | ICI | 0.06 | 0.05 | 1.18 | 0.258 |
|  |  | _cons | 0.56 | 0.10 | 5.41 | <0.001 |
|  | tumor type | tumor | 0.07 | 0.07 | 1.02 | 0.329 |
|  |  | _cons | 0.57 | 0.10 | 5.52 | <0.001 |
|  | NOS/Jadad score | score | 0.02 | 0.04 | 0.68 | 0.510 |
|  |  | _cons | 0.47 | 0.30 | 1.57 | 0.140 |
| OS for 12-month | year of publication | year | 0.07 | 0.03 | 2.12 | 0.050 |
|  |  | _cons | -136.77 | 64.66 | 2.12 | 0.050 |
|  | total sample | sample | 0.00 | 0.00 | 0.49 | 0.634 |
|  |  | _cons | 0.49 | 0.07 | 7.37 | 0.000 |
|  | number of center | center | 0.00 | 0.00 | 0.73 | 0.482 |
|  |  | _cons | 0.46 | 0.05 | 9.88 | 0.000 |
|  | ICI type | ICI | 0.12 | 0.05 | 2.33 | 0.033 |
|  |  | _cons | 0.24 | 0.11 | 2.23 | 0.041 |
|  | tumor type | tumor | 0.10 | 0.08 | 1.37 | 0.191 |
|  |  | _cons | 0.33 | 0.11 | 3.01 | 0.009 |
|  | NOS/Jadad score | score | 0.05 | 0.04 | 1.23 | 0.235 |
|  |  | _cons | 0.06 | 0.33 | 0.19 | 0.849 |
| OS for 24-month | year of publication | year | 0.08 | 0.04 | 2.10 | 0.060 |
|  |  | _cons | -169.14 | 80.79 | 2.09 | 0.060 |
|  | total sample | sample | 0.00 | 0.00 | 0.18 | 0.857 |
|  |  | _cons | 0.20 | 0.09 | 2.14 | 0.055 |
|  | number of center | center | 0.01 | 0.01 | 1.79 | 0.117 |
|  |  | _cons | 0.13 | 0.05 | 2.33 | 0.053 |
|  | ICI type | ICI | 0.11 | 0.07 | 1.51 | 0.160 |
|  |  | _cons | 0.02 | 0.14 | 0.14 | 0.892 |
|  | tumor type | tumor | 0.09 | 0.17 | 0.53 | 0.610 |
|  |  | _cons | 0.12 | 0.21 | 0.55 | 0.598 |
|  | NOS/Jadad score | score | 0.07 | 0.05 | 1.43 | 0.179 |
|  |  | _cons | -0.35 | 0.40 | 0.88 | 0.396 |
| PFS for 6-month | year of publication | year | 0.03 | 0.05 | 0.58 | 0.587 |
|  |  | _cons | -54.77 | 95.06 | 0.58 | 0.587 |
|  | total sample | sample | 0.00 | 0.00 | 0.68 | 0.529 |
|  |  | _cons | 0.29 | 0.12 | 2.46 | 0.057 |
|  | number of center | center | -0.23 | 0.06 | 0.49 | 0.656 |
|  |  | _cons | 0.36 | 0.13 | 2.83 | 0.066 |
|  | ICI type | ICI | 0.11 | 0.08 | 1.38 | 0.226 |
|  |  | _cons | 0.14 | 0.17 | 0.81 | 0.454 |
|  | tumor type | tumor | -0.23 | 0.19 | 1.24 | 0.269 |
|  |  | _cons | 0.61 | 0.22 | 2.82 | 0.037 |
|  | NOS/Jadad score | score | 0.08 | 0.06 | 1.32 | 0.244 |
|  |  | _cons | -0.32 | 0.51 | 0.62 | 0.562 |
| PFS for 12-month | year of publication | year | 0.05 | 0.04 | 1.20 | 0.298 |
|  |  | _cons | -107.69 | 90.24 | 1.19 | 0.299 |
|  | total sample | sample | 0.00 | 0.00 | 0.07 | 0.948 |
|  |  | _cons | 0.30 | 0.14 | 2.05 | 0.110 |
|  | number of center | center | -0.03 | 0.02 | 1.42 | 0.291 |
|  |  | _cons | 0.31 | 0.06 | 4.81 | 0.041 |
|  | ICI type | ICI | 0.10 | 0.08 | 1.30 | 0.265 |
|  |  | _cons | 0.08 | 0.17 | 0.45 | 0.676 |
|  | tumor type | tumor | / | / | / | / |
|  |  | _cons | 0.29 | 0.07 | 4.15 | 0.009 |
|  | NOS/Jadad score | score | 0.07 | 0.06 | 1.12 | 0.326 |
|  |  | _cons | -0.35 | 0.58 | 0.61 | 0.575 |
| PFS for 24-month | year of publication | year | 0.09 | 0.05 | 1.86 | 0.159 |
|  |  | _cons | -180.33 | 96.95 | 1.86 | 0.160 |
|  | total sample | sample | 0.00 | 0.00 | 0.28 | 0.797 |
|  |  | _cons | 0.26 | 0.19 | 1.43 | 0.249 |
|  | number of center | center | -0.14 | 0.25 | 0.59 | 0.662 |
|  |  | _cons | 0.38 | 0.34 | 1.10 | 0.469 |
|  | ICI type | ICI | 0.21 | 0.07 | 3.10 | 0.053 |
|  |  | _cons | -0.17 | 0.13 | 1.28 | 0.289 |
|  | tumor type | tumor | / | / | / | / |
|  |  | _cons | 0.22 | 0.09 | 2.37 | 0.077 |
|  | NOS/Jadad score | score | 0.13 | 0.07 | 1.88 | 0.157 |
|  |  | _cons | -0.94 | 0.62 | 1.52 | 0.227 |

Abbreviation: Coef.: Coefficient; Std. Err.: Standard Error; OS: Overall Survival; PFS: Progression Free Survival; ICI: Immune checkpoint inhibitor; NOS: Newcastle-Ottawa Scale.
