# Supplementary material for: Immune Checkpoint Inhibitors and Survival Outcomes in Brain Metastasis: A Time Series-Based Meta-Analysis
Source: Front Oncol. 2020 Oct 20;10:564382. doi: 10.3389/fonc.2020.564382 (PMC7606910; doi:10.3389/fonc.2020.564382)
Supplement: Supplementary file 1 [file Data_Sheet_1.zip › Supplementary materials/Supplementary Table 2 Beggs test for each outcome of included studies.docx]

**Supplementary table 2. Begg’s test for each outcome of included studies**

|  | **OS for 6-month** | **OS for 12-month** | **OS for 24-month** | **PFS for 6-month** | **PFS for 12-month** | **PFS for 24-month** |
| --- | --- | --- | --- | --- | --- | --- |
| No. of studies | 16 | 18 | 14 | 7 | 6 | 5 |
| *Z* | 0.23 | 0.68 | 0.62 | 1.22 | 0.75 | 0.73 |
| *P* | 0.822 | 0.495 | 0.536 | 0.221 | 0.452 | 0.462 |

Abbreviation: OS: Overall Survival; PFS: Progression Free Survival.
