# Supplementary material for: Immune Checkpoint Inhibitors and Survival Outcomes in Brain Metastasis: A Time Series-Based Meta-Analysis
Source: Front Oncol. 2020 Oct 20;10:564382. doi: 10.3389/fonc.2020.564382 (PMC7606910; doi:10.3389/fonc.2020.564382)
Supplement: Supplementary file 1 [file Data_Sheet_1.zip › Supplementary materials/Supplementary Table 3 Eggers test for each outcome of included studies.docx]

**Supplementary table 3. Egger’s test for each outcome of included studies**

| **Outcome** |  | **Coef.** | **Std. Err.** | ***t*** | ***P*** |
| --- | --- | --- | --- | --- | --- |
| OS for 6-month | slope | 0.79 | 0.10 | 8.03 | <0.001 |
|  | bias | -1.76 | 1.76 | 1.00 | 0.336 |
| OS for 12-month | slope | 0.41 | 0.12 | 3.37 | 0.004 |
|  | bias | 0.78 | 1.96 | 0.40 | 0.697 |
| OS for 24-month | slope | -0.03 | 0.08 | 0.32 | 0.757 |
|  | bias | 4.63 | 2.22 | 2.08 | 0.062 |
| PFS for 6-month | slope | 0.40 | 0.16 | 2.44 | 0.050 |
|  | bias | -0.83 | 2.71 | 0.31 | 0.770 |
| PFS for 12-month | slope | 0.14 | 0.17 | 0.79 | 0.476 |
|  | bias | 2.53 | 3.46 | 0.73 | 0.505 |
| PFS for 24-month | slope | -0.03 | 0.09 | -0.29 | 0.789 |
|  | bias | 4.66 | 2.88 | 1.62 | 0.204 |

Abbreviation: Coef.: Coefficient; Std. Err.: Standard Error; OS: Overall Survival; PFS: Progression Free Survival.
