# Supplementary material for: Immune Checkpoint Inhibitors and Survival Outcomes in Brain Metastasis: A Time Series-Based Meta-Analysis
Source: Front Oncol. 2020 Oct 20;10:564382. doi: 10.3389/fonc.2020.564382 (PMC7606910; doi:10.3389/fonc.2020.564382)
Supplement: Supplementary file 1 [file Data_Sheet_1.zip › Supplementary materials/Supplementary Table 4 Media OS and media PFS reported as the abstract on the academic conference.docx]

**Supplementary table 4. Media OS and media PFS reported as the abstract on the academic conference**

| **Study** | **Trial** | **Disease** | **Patients**  **(n)** | **ICI Target** | **mOS, months (95%CI)** | **mPFS, months (95%CI)** |
| --- | --- | --- | --- | --- | --- | --- |
| Jonathan et al. 2016 | Checkmate 063/017/057 | NSCLC | 46 | PD-1 | 8.4 (5.0, 11.5) | NR |
| Molinier et al. 2017 | French EAP | NSCLC | 130 | PD-1 | 9.5 (8.4, 10.8) | 2.1 (1.9, 2.3) |
| Crino et al. 2017 | Italian EAP | NSCLC | 409 | PD-1 | 8.1(6.2, 10.1) | NR |
| Lauko et al. 2018 | NR | NSCLC | 94 | ICI | 28.9 | 3.6 |
|  |  | RCC | 15 | ICI | NR | 5.9 |
|  |  | melanoma | 19 | ICI | 17.1 | 6.7 |
| Karivedu et al. 2018 | NR | NSCLC | 24 | CTLA-4/PD-1/PD-L1 | 7.2 | 2.0 |
|  |  | melanoma | 18 | CTLA-4/PD-1/PD-L1 | 7.6 | 5.1 |

**Abbreviation:**  ICI: Immune checkpoint inhibitors; mOS: Media overall survival; mPFS: Media progression-free survival; NSCLC: Non-small cell carcinoma; NR: Not report; PD-1: Programmed cell death-1; PD-L1: Programmed cell death ligand 1; CTLA-4: anti-cytotoxic T-lymphocyte-associated protein 4.

**References:**

[1] Jonathan et al. 2016: Jonathan WG, Lucio C, Everett EV, Esther H, Karen R, Adam P, et al. Nivolumab in patients with advanced NSCLC and central nervous system metastases [Abstract]. *J Thorac Oncol.* (2016), 11(10S): S238-9. P2.36.

[2] Molinier et al. 2017: Molinier O, Audigier-Valette C, Cadranel J, Monnet I, Hureaux J, Hilgers W, et al. Real-life experience with Nivolumab in 600 patients with advanced Non-Small Cell Lung Cancer [Abstract]. IASLC 18^th^ WCLC: 137. OA 17.05.

[3] Crino et al. 2017: Crino L, Bidoli P, Ulivi P, Minenza E, Cortesi E, Garassino M, et al. Italian Nivolumab expanded access programme: Data from patients with advanced Non-Squamous NSCLC and brain metastases [Abstract]. IASLC 18^th^ WCLC: 137. P1.01-053.

[4] Lauko et al. 2018: Lauko A, Thapa B, Jia X, Ahluwalia MS. Efficacy of immune checkpoint inhibitors in patients with brain metastasis from NSCLC, RCC, and melanoma [Abstract]. *J Clin Oncol*. (2018) 36:214. Doi: 10.1200/JCO.2018.36.5_supple.214

[5] Karivedu et al. 2018: Karivedu V, Jandarov R, Wise-Draper TM. Brain metastases treated with immune checkpoint inhibitors: a single center experience [Abstract]. *J Clin Oncol*. (2018) 36: e14012. Doi: 10.1200/JCO.2018.36.15_suppl.e14012
