# Supplementary material for: Immune Checkpoint Inhibitors and Survival Outcomes in Brain Metastasis: A Time Series-Based Meta-Analysis
Source: Front Oncol. 2020 Oct 20;10:564382. doi: 10.3389/fonc.2020.564382 (PMC7606910; doi:10.3389/fonc.2020.564382)
Supplement: Supplementary file 1 [file Data_Sheet_1.zip › Supplementary materials/Supplementary Table 5 Characteristics of prospective studies under research (Recruiting and Active).docx]

**Supplementary table 5 Characteristics of prospective studies under research (Recruiting and Active)**

| Trial | Intervention | Disease | Phase | Enrollment(n) | Allocation | Period |
| --- | --- | --- | --- | --- | --- | --- |
| NCT02460068  (NIBIT-M2) | • Drug: Fotemustine  • Drug: Fotemustine+Ipilimumab  • Drug: Ipilimumab+nivolumab | • Brain Metastases | 3 | 168 | Randomized | 2012.12-2020.01 |
| NCT02374242  (ABC) | • Drug: Nivolumab  • Drug: Ipilimumab | • Melanoma  • Brain Metastases | 2 | 76 | Randomized | 2014.11-2022.12 |
| NCT02886585 | • Drug: Pembrolizumab  • Radiation: MRI  • Radiation: PET/CT  • Procedure: Stereotactic Radiosurgery | • Brain Metastases | 2 | 102 | Non-Randomized | 2016.10-2024.01 |
| NCT03728465 | • Biological: Nivolumab  • Biological: Ipilimumab | • Metastatic Melanoma  • Brain Metastases | 2 | 68 | Single Group Assignment | 2018.04-2024.09 |
| NCT03563729  (MEMBRAINS) | • Drug: Pembrolizumab  • Drug: Ipilimumab  • Drug: Nivolumab | • Metastatic Melanoma | 2 | 80 | Non-Randomized | 2018.06-2025.06 |
