# Supplementary material for: Immune Checkpoint Inhibitors and Survival Outcomes in Brain Metastasis: A Time Series-Based Meta-Analysis
Source: Front Oncol. 2020 Oct 20;10:564382. doi: 10.3389/fonc.2020.564382 (PMC7606910; doi:10.3389/fonc.2020.564382)
Supplement: Supplementary file 1 [file Data_Sheet_1.zip › Supplementary materials/Supplementary Table 6. Treatment-related adverse events of including studies.docx]

Supplementary tables 6**. Treatment-related adverse events of including studies**

| **AE name** | **Study** | **ICI target** | **Drug designation** | **Any grade** | **Grade 1/2** | **Grade 3/4** |
| --- | --- | --- | --- | --- | --- | --- |
| Colitis | G. V. Long et al., 2018 | Combined CTLA-4 and PD-1/PD-L1 | Nivolumab plus ipilimumab | 28 (36.8) | 21 (27.6) | 7 (9.2) |
|  | H. A. Tawbi et al., 2018 | Combined CTLA-4 and PD-1/PD-L1 | Nivolumab plus ipilimumab | 7 (7) | 0 | 7 (7) |
|  | K. R. Patel et al., 2017 | CTLA-4 | Ipilimumab | 1 (3.2) | / | / |
| Diarrhea | C. Gauvain et al., 2018 | PD-1/PD-L1 | Nivolumab | 6 (14) | / | 2 (5) |
|  | H. M. Kluger et al., 2018 | PD-1/PD-L1 | Pembrolizumab | 2 (9) | 2 (9) | 0 |
|  | H. A. Tawbi et al., 2018 | Combined CTLA-4 and PD-1/PD-L1 | Nivolumab plus ipilimumab | 33 (35) | / | 6 (6) |
|  | P. Queirolo et al., 2014 | CTLA-4 | Ipilimumab | 14 (10) | / | 2 (1) |
| Dry mouth | G. V. Long et al., 2018 | Combined CTLA-4 and PD-1/PD-L1 | Nivolumab plus ipilimumab | 7 (9.2) | 7 (9.2) | / |
|  | H. M. Kluger et al., 2018 | PD-1/PD-L1 | Pembrolizumab | 1 (4) | 1 (4) | 0 |
| Fatigue | G. V. Long et al., 2018 | Combined CTLA-4 and PD-1/PD-L1 | Nivolumab plus ipilimumab | 31 (40.8) | 30 (39.5) | 1 (1.3) |
|  | H. A. Tawbi et al., 2018 | Combined CTLA-4 and PD-1/PD-L1 | Nivolumab plus ipilimumab | 45 (48) | / | 4 (4) |
| Headache | G. V. Long et al., 2018 | Combined CTLA-4 and PD-1/PD-L1 | Nivolumab plus ipilimumab | 10 (13.2) | 9 (11.8) | 1 (1.3) |
|  | H. A. Tawbi et al., 2018 | Combined CTLA-4 and PD-1/PD-L1 | Nivolumab plus ipilimumab | 21 (22) | / | 3 (3) |
|  | P. Queirolo et al., 2014 | CTLA-4 | Ipilimumab | 1 (1) | 0 | 1 (1) |
| Liver toxicity | C. Gauvain et al., 2018 | PD-1/PD-L1 | Nivolumab | 1 (2) | / | / |
|  | G. V. Long et al., 2018 | Combined CTLA-4 and PD-1/PD-L1 | Nivolumab plus ipilimumab | 23 (30.3) | 14 (18.4) | 9 (11.8) |
|  | P. Queirolo et al., 2014 | CTLA-4 | Ipilimumab | 4 (3) | 0 | 4 (3) |
| Nausea | H. A. Tawbi et al., 2018 | Combined CTLA-4 and PD-1/PD-L1 | Nivolumab plus ipilimumab | 26 (28) | / | 2 (2) |
|  | P. Queirolo et al., 2014 | CTLA-4 | Ipilimumab | 7 (5) | / | 1 (1) |
| Pruritus | G. V. Long et al., 2018 | Combined CTLA-4 and PD-1/PD-L1 | Nivolumab plus ipilimumab | 17 (22.4) | 17 (22.4) | 0 |
|  | H. M. Kluger et al., 2018 | PD-1/PD-L1 | Pembrolizumab | 5 (22) | 5 (22) | 0 |
|  | P. Queirolo et al., 2014 | CTLA-4 | Ipilimumab | 4 (3) | / | / |
| Rash | G. V. Long et al., 2018 | Combined CTLA-4 and PD-1/PD-L1 | Nivolumab plus ipilimumab | 30 (39.5) | 26 (34.2) | 4 (5.3) |
|  | H. M. Kluger et al., 2018 | PD-1/PD-L1 | Pembrolizumab | 6 (26) | 5 (22) | 1 (4) |
|  | H. A. Tawbi et al., 2018 | Combined CTLA-4 and PD-1/PD-L1 | Nivolumab plus ipilimumab | 8 (9) | / | 2 (2) |
|  | P. Queirolo et al., 2014 | CTLA-4 | Ipilimumab | 4 (3) | / | / |
| Vomiting | G. V. Long et al., 2018 | Combined CTLA-4 and PD-1/PD-L1 | Nivolumab plus ipilimumab | 19 (25) | 18 (23.7) | 1 (1.3) |
|  | H. A. Tawbi et al., 2018 | Combined CTLA-4 and PD-1/PD-L1 | Nivolumab plus ipilimumab | 12 (13) | / | 2 (2) |
|  | P. Queirolo et al., 2014 | CTLA-4 | Ipilimumab | 5 (3) | / | 1 (1) |

Abbreviation: ACTH: Adrenocorticotropic hormone ; CTLA-4: anti-cytotoxic T-lymphocyte-associated protein 4; ICI: Immune checkpoint inhibitors; PD-1: Programmed cell death-1; PD-L1: Programmed cell death ligand 1.
