# Supplementary material for: Immune Checkpoint Inhibitors and Survival Outcomes in Brain Metastasis: A Time Series-Based Meta-Analysis
Source: Front Oncol. 2020 Oct 20;10:564382. doi: 10.3389/fonc.2020.564382 (PMC7606910; doi:10.3389/fonc.2020.564382)
Supplement: Supplementary file 1 [file Data_Sheet_1.zip › Supplementary materials/Captions of Supplementary files.docx]

**Supplementary Figure 1**

Forest plots showing 6-month PFS in BM patients treated with ICIs according to tumor type

**Supplementary Figure 2**

Forest plots showing 6-month PFS in BM patients treated with ICIs according to ICI type

**Supplementary Figure 3**

Forest plots showing 12-month PFS in BM patients treated with ICIs according to tumor type

**Supplementary Figure 4**

Forest plots showing 12-month PFS in BM patients treated with ICIs according to ICI type

**Supplementary Figure 5**

Forest plots showing 6-month survival rate in BM patients treated with ICIs according to tumor type

**Supplementary Figure 6**

Forest plots showing 6-month survival rate in BM patients treated with ICIs according to ICI type.

**Supplementary Figure 7**

Forest plots showing 12-month survival rate in BM patients treated with ICIs according to tumor type

**Supplementary Figure 8**

Forest plots showing 12-month survival rate in BM patients treated with ICIs according to ICI type

**Supplementary Figure 9**

Forest plots showing 24-month PFS in BM patients treated with ICIs

**Supplementary Figure 10**

Forest plots showing 24-month survival rate in BM patients treated with ICIs

**Supplementary Figure 11**

Sensitivity plots for the main results in BM patients treated with ICIs

6-month survival rate(A), 6-month PFS(B), 12-month survival rate(C), 12-month PFS(D), 24-month survival rate(E), 6-month PFS(F)

**Supplementary Figure 12**

Funnel plots for publication bias with studies concerning treatment responses in BM patients treated with ICIs

6-month survival rate(A), 6-month PFS(B), 12-month survival rate(C), 12-month PFS(D), 24-month survival rate(E), 6-month PFS(F)
