# Supplementary figures and images for: Immune Checkpoint Inhibitors and Survival Outcomes in Brain Metastasis: A Time Series-Based Meta-Analysis
Source: Front Oncol. 2020 Oct 20;10:564382. doi: 10.3389/fonc.2020.564382 (PMC7606910; doi:10.3389/fonc.2020.564382)

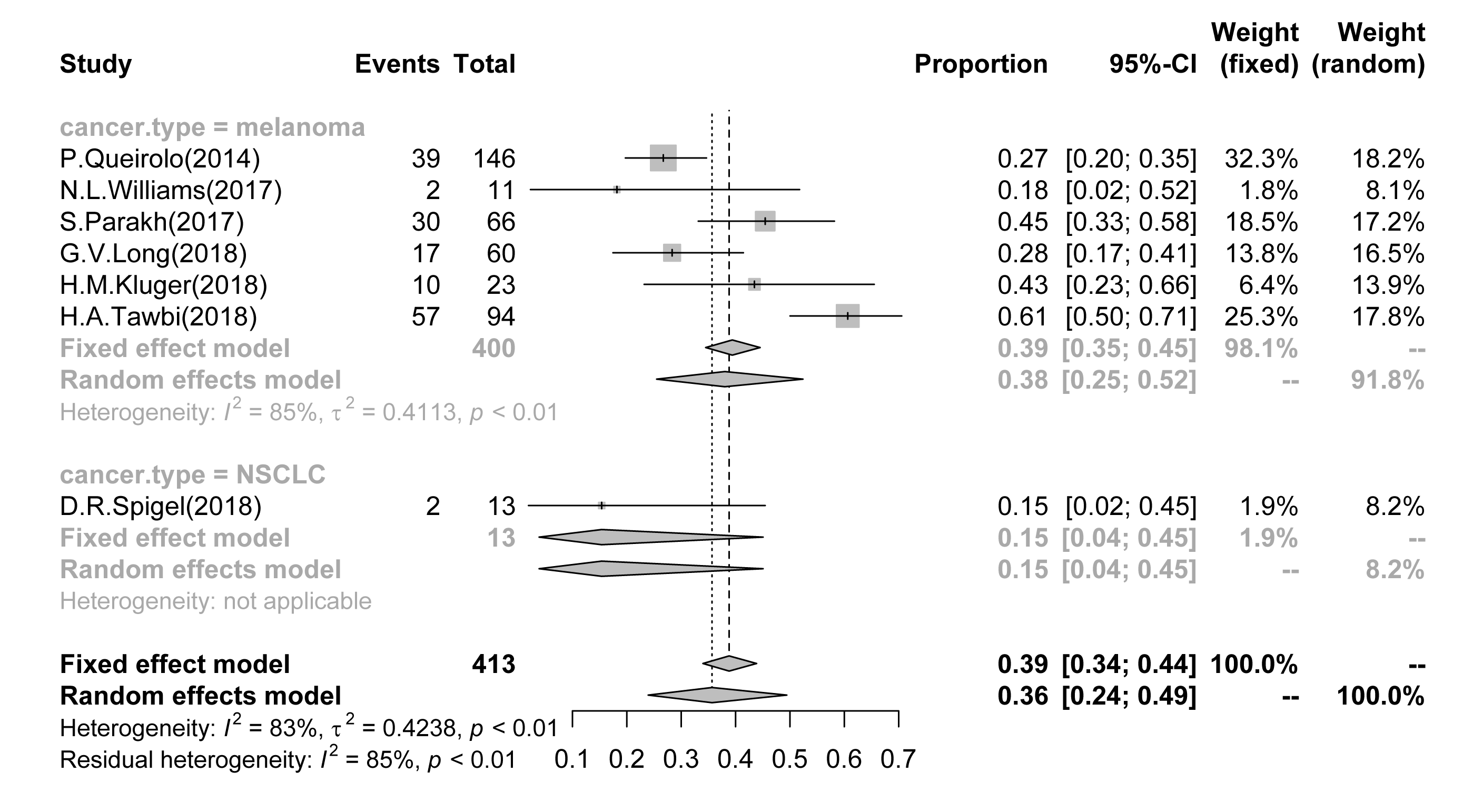

Supplement: Supplementary file 1 [file Data_Sheet_1.zip › Supplementary materials/Supplementary Figure 1 PFS_6mo_forest_tumor.tif]

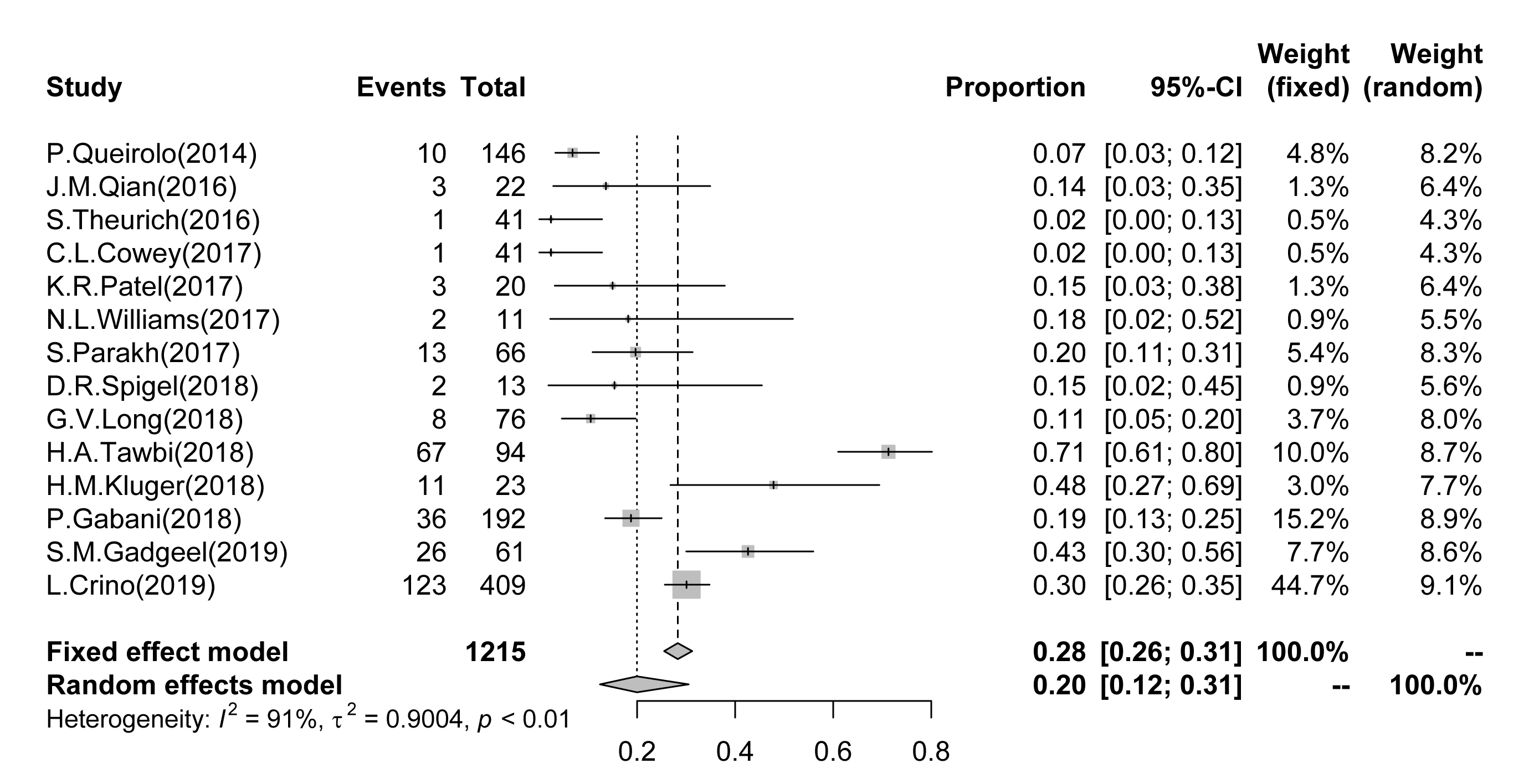

Supplement: Supplementary file 1 [file Data_Sheet_1.zip › Supplementary materials/Supplementary Figure 10 OS_24mo_forest.tif]

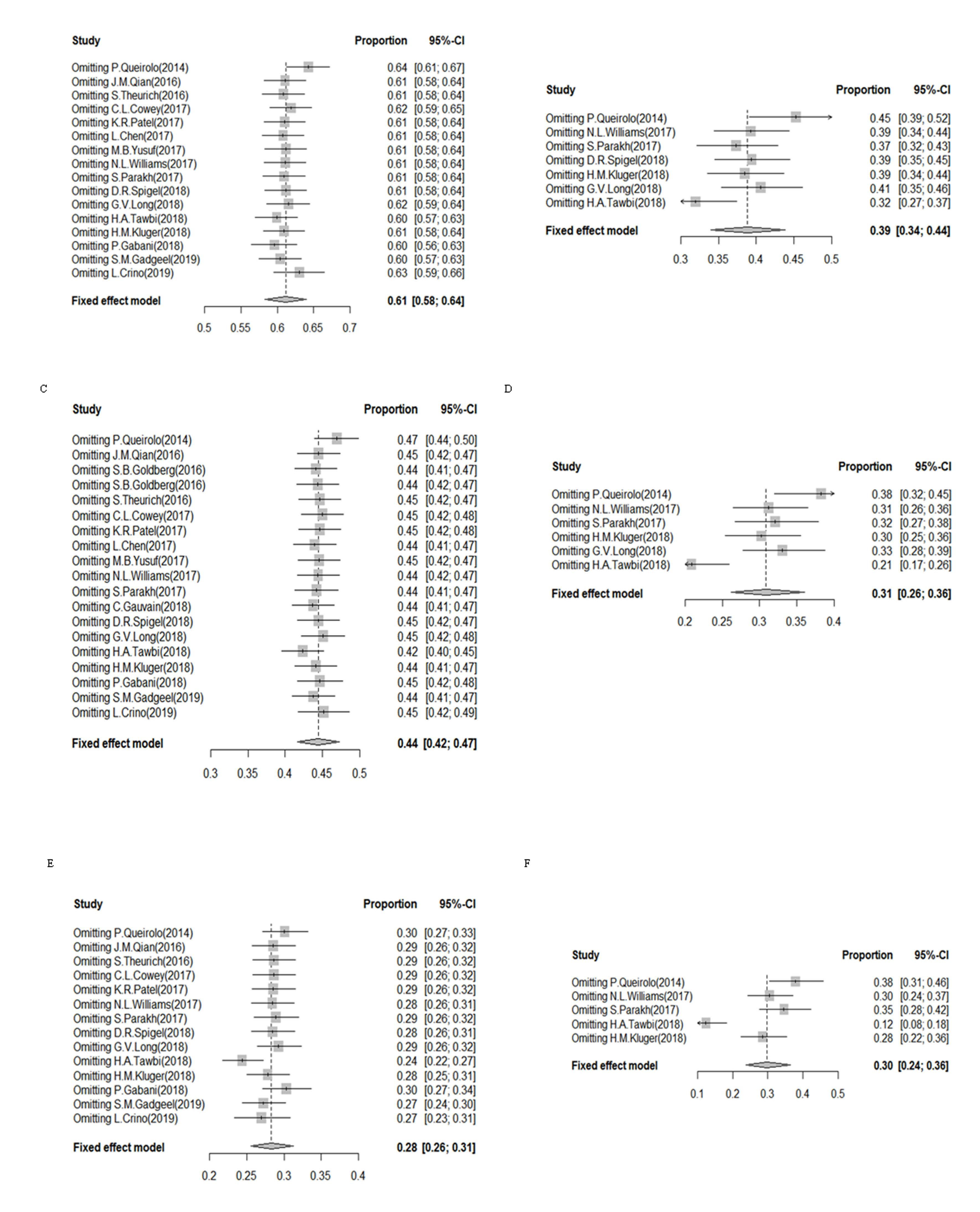

Supplement: Supplementary file 1 [file Data_Sheet_1.zip › Supplementary materials/Supplementary Figure 11 metainf plot.tif]

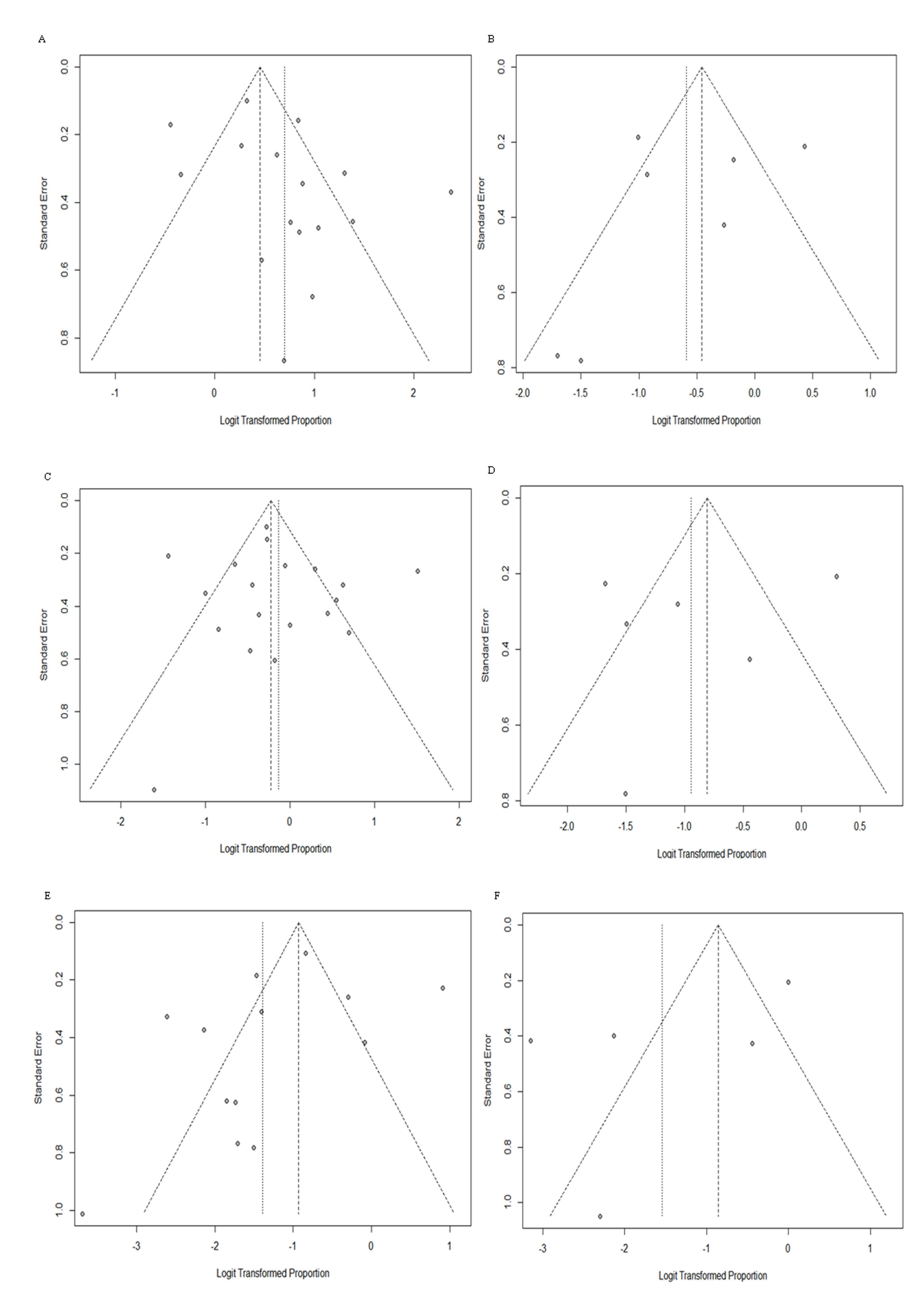

Supplement: Supplementary file 1 [file Data_Sheet_1.zip › Supplementary materials/Supplementary Figure 12 funnel plot.tif]

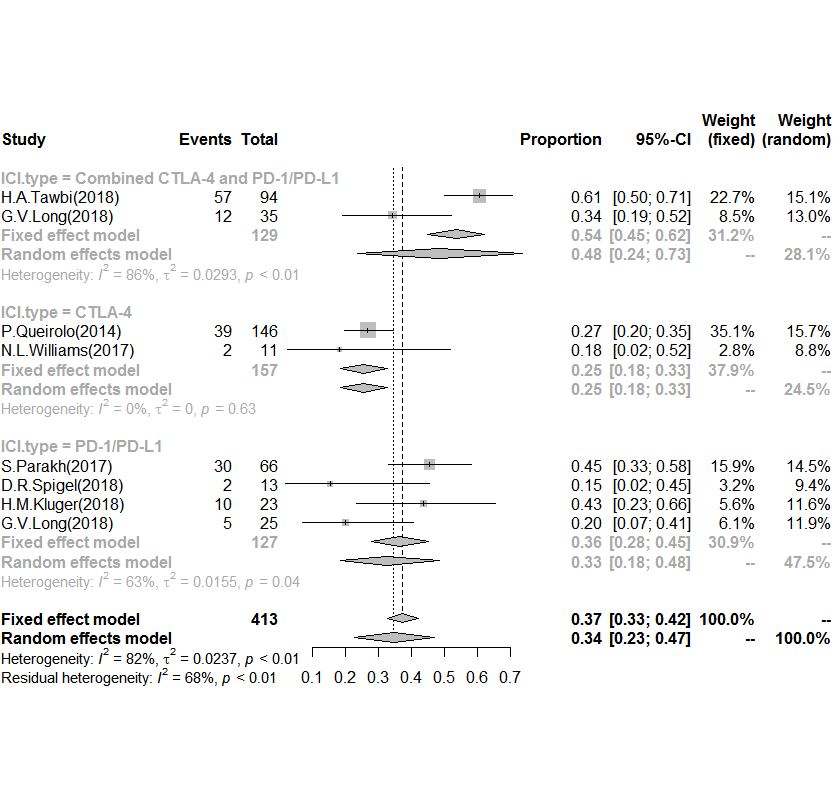

Supplement: Supplementary file 1 [file Data_Sheet_1.zip › Supplementary materials/Supplementary Figure 2 PFS_6mo_forest_ICI.tiff]

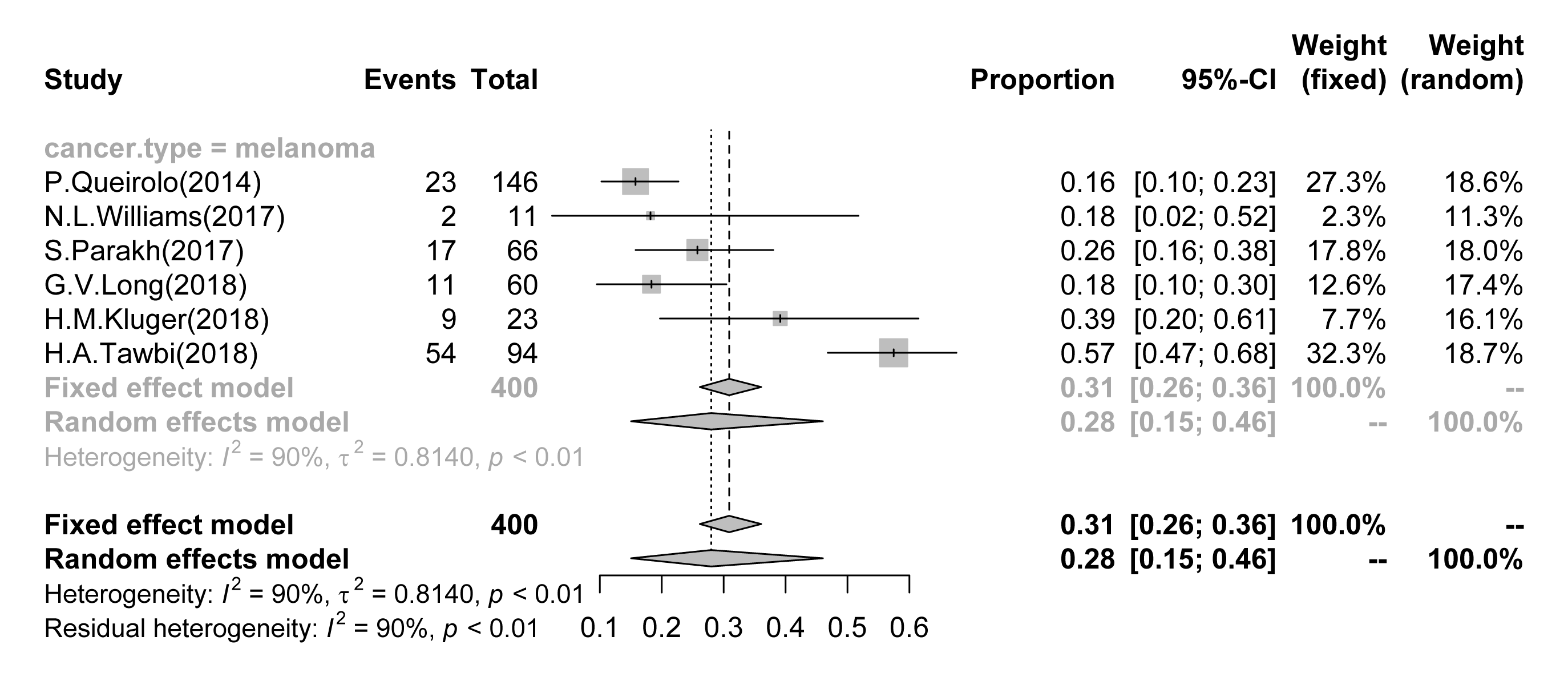

Supplement: Supplementary file 1 [file Data_Sheet_1.zip › Supplementary materials/Supplementary Figure 3 PFS_12mo_forest_tumor.tif]

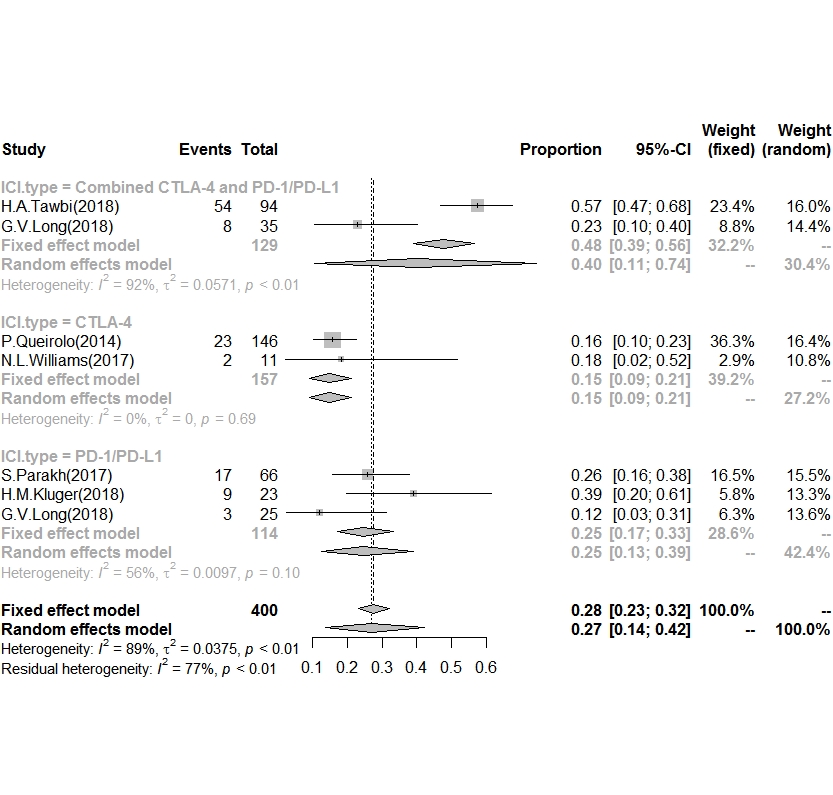

Supplement: Supplementary file 1 [file Data_Sheet_1.zip › Supplementary materials/Supplementary Figure 4 PFS_12mo_forest_ICI.tiff]

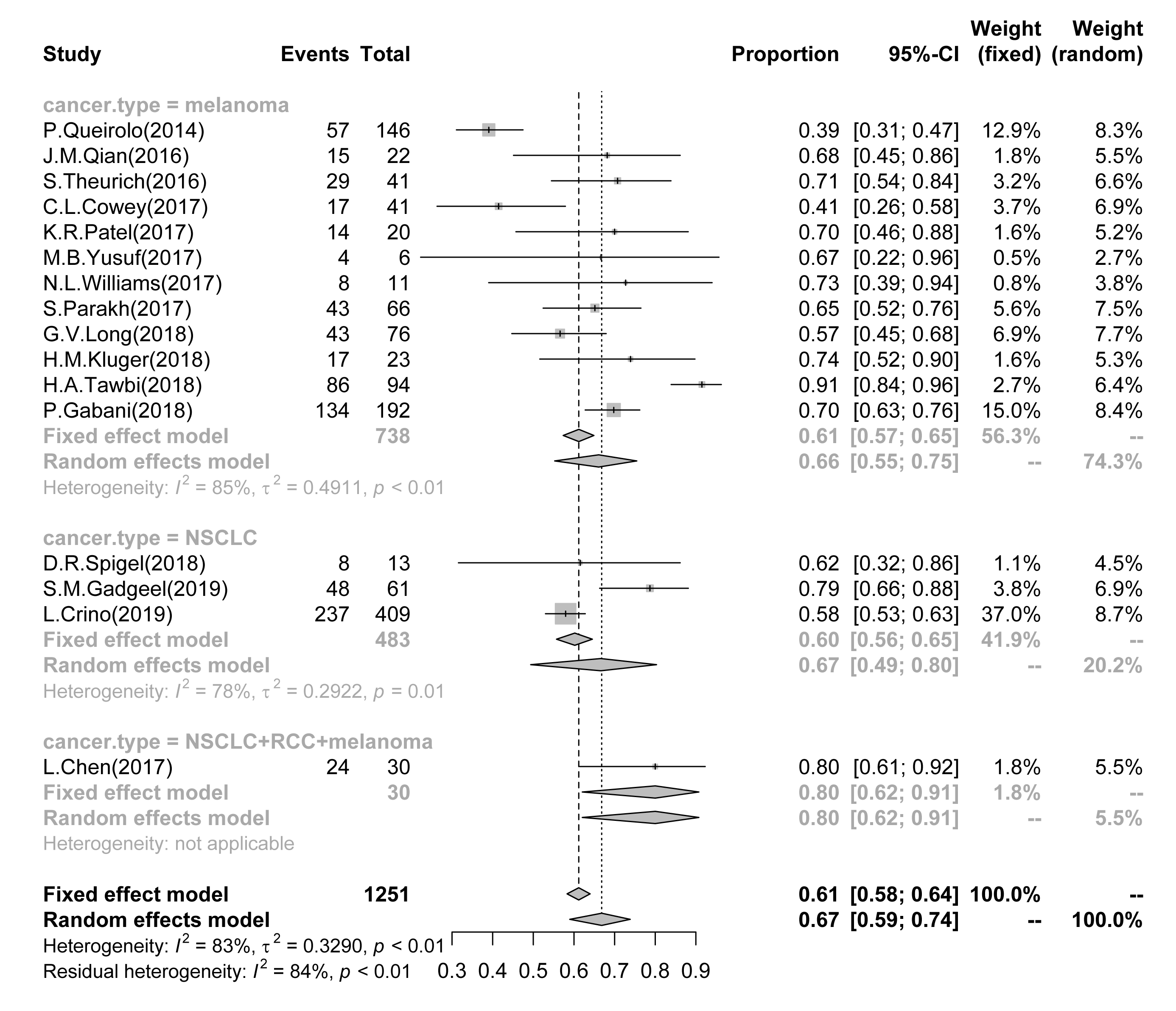

Supplement: Supplementary file 1 [file Data_Sheet_1.zip › Supplementary materials/Supplementary Figure 5 OS_6mo_forest_tumor.tif]

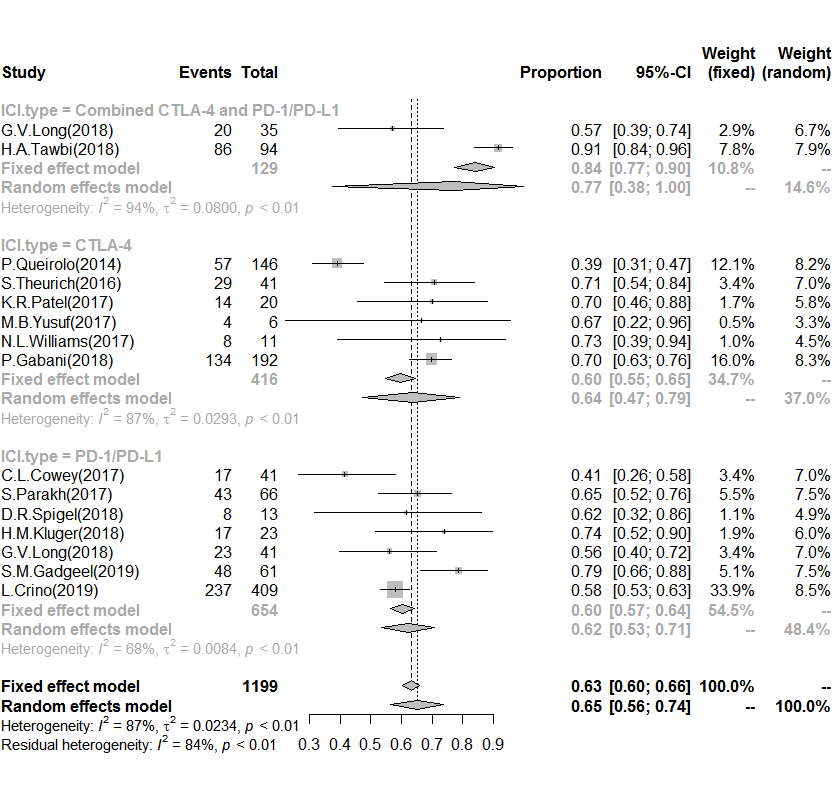

Supplement: Supplementary file 1 [file Data_Sheet_1.zip › Supplementary materials/Supplementary Figure 6 OS_6mo_forest_ICI.tiff]

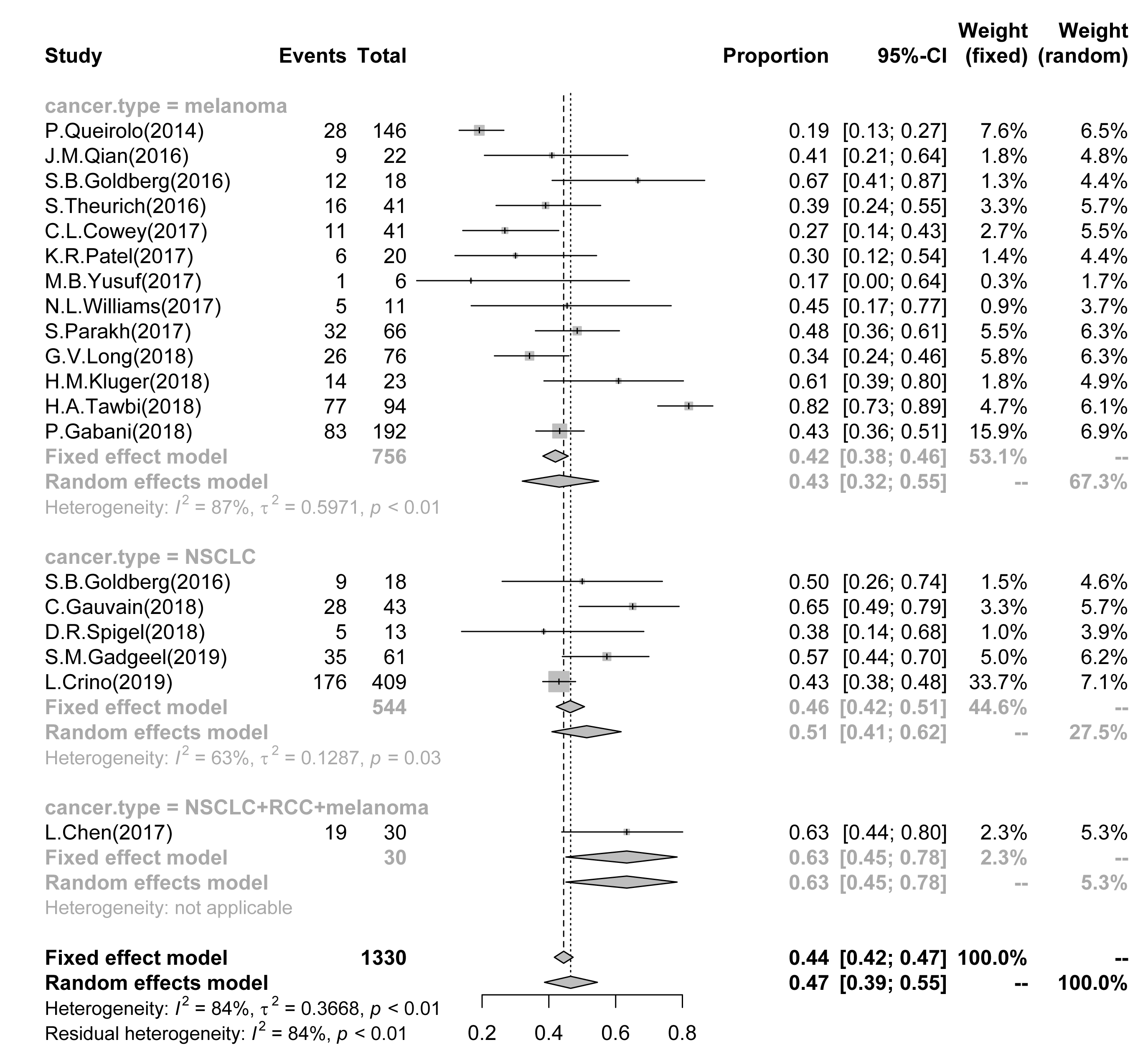

Supplement: Supplementary file 1 [file Data_Sheet_1.zip › Supplementary materials/Supplementary Figure 7 OS_12mo_forest_tumor.tif]

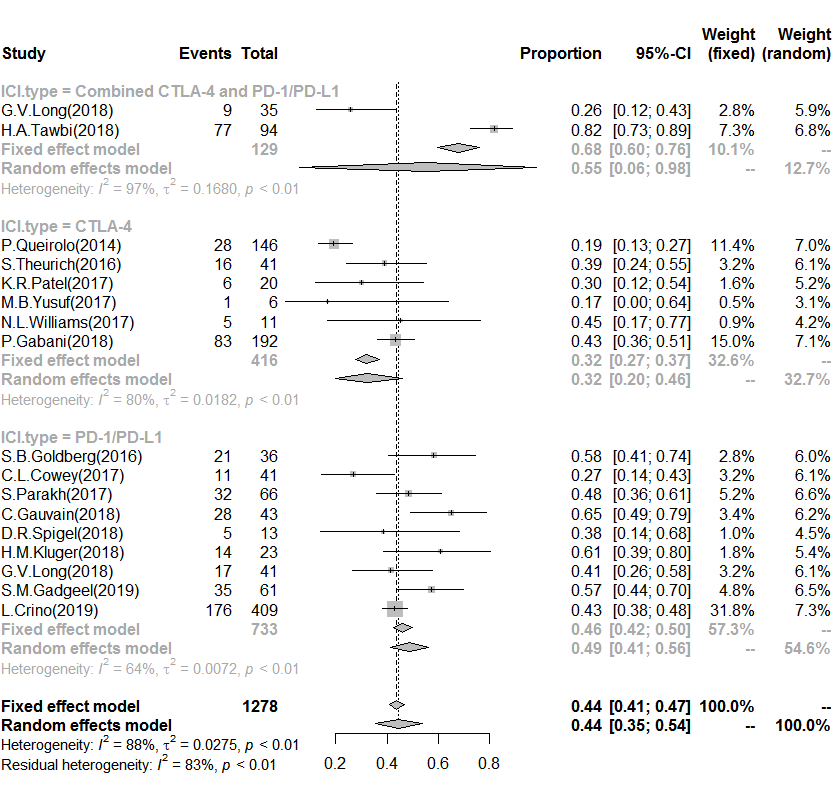

Supplement: Supplementary file 1 [file Data_Sheet_1.zip › Supplementary materials/Supplementary Figure 8 OS_12mo_forest_ICI.tiff]

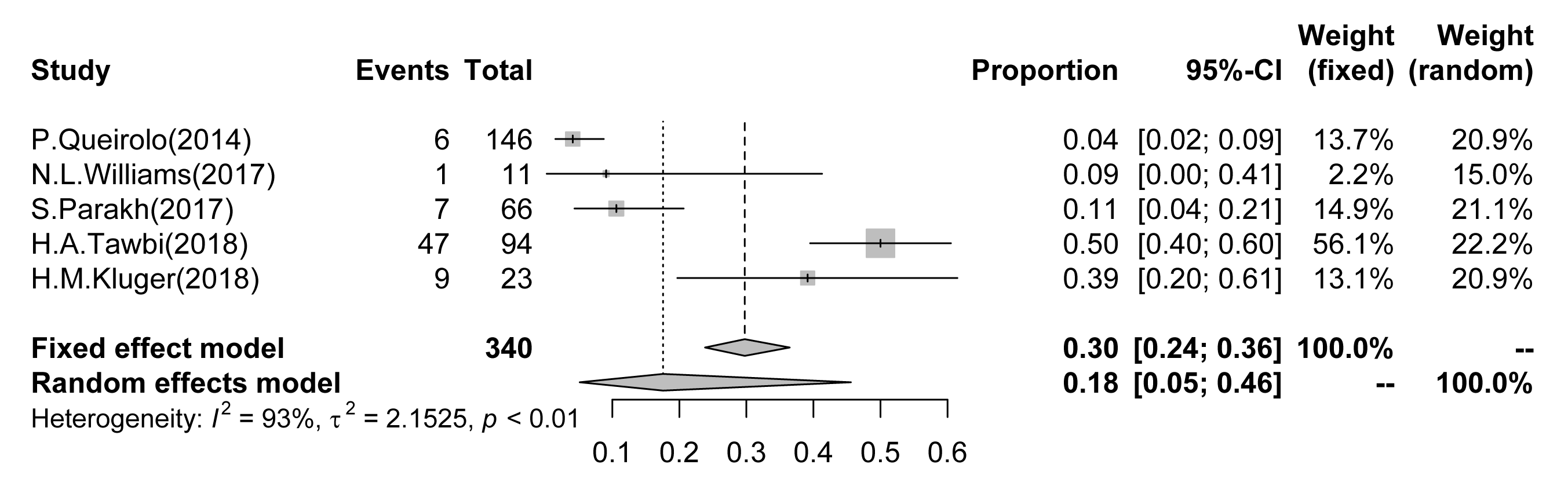

Supplement: Supplementary file 1 [file Data_Sheet_1.zip › Supplementary materials/Supplementary Figure 9 PFS_24mo_forest.tif]
